# Intermittent glucocorticoid treatment enhances skeletal muscle performance through sexually dimorphic mechanisms

**DOI:** 10.1172/JCI149828

**Published:** 2022-03-15

**Authors:** Isabella M. Salamone, Mattia Quattrocelli, David Y. Barefield, Patrick G. Page, Ibrahim Tahtah, Michele Hadhazy, Garima Tomar, Elizabeth M. McNally

**Affiliations:** 1 Center for Genetic Medicine, Northwestern University, Chicago, Illinois, USA.; 2 Molecular Cardiovascular Biology Institute, Cincinnati Children’s Hospital Medical Center and Department of Pediatrics, University of Cincinnati College of Medicine, Cincinnati, Ohio, USA.; 3 Cell and Molecular Physiology, Loyola University Chicago, Maywood, Illinois, USA.; 4 Department of Physiology, Vrije Universiteit Amsterdam, Amsterdam, Netherlands.

**Keywords:** Endocrinology, Muscle Biology, Calcium, Insulin signaling, Skeletal muscle

## Abstract

Glucocorticoid steroids are commonly prescribed for many inflammatory conditions, but chronic daily use produces adverse effects, including muscle wasting and weakness. In contrast, shorter glucocorticoid pulses may improve athletic performance, although the mechanisms remain unclear. Muscle is sexually dimorphic and comparatively little is known about how male and female muscles respond to glucocorticoids. We investigated the impact of once-weekly glucocorticoid exposure on skeletal muscle performance comparing male and female mice. One month of once-weekly glucocorticoid dosing improved muscle specific force in both males and females. Transcriptomic profiling of isolated myofibers identified a striking sexually dimorphic response to weekly glucocorticoids. Male myofibers had increased expression of genes in the IGF1/PI3K pathway and calcium handling, while female myofibers had profound upregulation of lipid metabolism genes. Muscles from weekly prednisone–treated males had improved calcium handling, while comparably treated female muscles had reduced intramuscular triglycerides. Consistent with altered lipid metabolism, weekly prednisone–treated female mice had greater endurance relative to controls. Using chromatin immunoprecipitation, we defined a sexually dimorphic chromatin landscape after weekly prednisone. These results demonstrate that weekly glucocorticoid exposure elicits distinct pathways in males versus females, resulting in enhanced performance.

## Graphical abstract

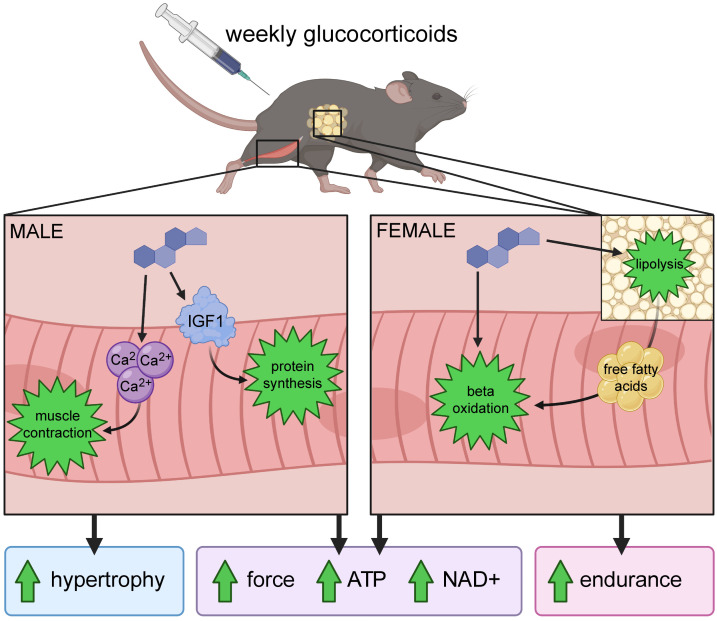

## Introduction

Glucocorticoids are powerful stress hormones that modulate the body’s carbohydrate, lipid, and protein metabolism in response to external or circadian stimuli. Synthetic glucocorticoids such as prednisone are used as antiinflammatory agents for conditions ranging from rheumatoid arthritis to muscular dystrophy to asthma. The therapeutic benefits of glucocorticoids are hampered by extensive side effects especially when taken chronically, and these side effects include insulin resistance and muscle weakness (1). Chronic glucocorticoid treatment is known to induce skeletal muscle atrophy by stimulating atrogene expression (2, 3), and despite this, glucocorticoids are still widely used because of their effectiveness as antiinflammatory agents.

Glucocorticoids exert their effects through the glucocorticoid receptor (GR). Upon ligand binding, GR is released from a chaperone complex in the cytoplasm and transported into the nucleus, where it binds glucocorticoid-response elements (GREs) near target genes. GR interacts with a diverse group of coactivators and corepressors in order to regulate target gene expression in a tissue- and stimulus-specific manner (4, 5). The role of GR as an antiinflammatory transcription factor is well delineated (6, 7). In muscle, glucocorticoids and GR have been well characterized in the promotion of skeletal muscle atrophy (3, 8–10). In high or chronic dosing schemes, glucocorticoids activate the ubiquitin proteasome pathway, upregulating the muscle-specific ubiquitin ligases MuRF-1 and atrogin-1, which directly increase polyubiquitination-mediated degradation of proteins and initiate muscle wasting (11, 12). Concurrently, these high doses of glucocorticoids inhibit mammalian target of rapamycin (mTOR) (13–15), resulting in decreased protein synthesis.

Glucocorticoids have been used to enhance muscle performance and/or improve recovery from injury, often in short dosing regimens thought to act through antiinflammatory properties (16). Studies examining lower dose and short-term exposure to glucocorticoids have found inconsistent results in skeletal muscle performance, likely related to varying dosing schemes. A single dose or short-term dosing, for less than 1 week, has been found to result in either no change or modest improvements in athletic performance in both humans (16) and mice (17, 18). In mice, a chronic once-weekly dosing regimen improved muscle strength and endurance in models of Duchenne muscular dystrophy (DMD) and limb girdle muscular dystrophies 2B and 2C (19, 20). The molecular mechanisms responsible for this benefit to muscle are likely multifold, and studies in mouse models of DMD indicate that the transcription factor KLF15 is relevant in the diseased muscle setting (17, 20). Few of the studies examining glucocorticoid dosing and response have considered sex-specific responses, despite skeletal muscle being a highly sexually dimorphic tissue and the observations of sex-specific transcriptional programs driven by glucocorticoids in other tissues (21).

To investigate how prednisone dosing impacts atrogene expression and muscle performance, we treated a cohort of male and female adult mice with once-weekly or daily doses of prednisone for 1 month and then evaluated skeletal muscle function and gene expression. We found that daily prednisone treatment resulted in decreased specific force and increased atrogene expression in both males and females. In contrast, once-weekly prednisone–treated mice of both sexes exhibited increased specific force, increased skeletal muscle ATP, and no increase in atrogene expression. When we investigated the skeletal muscle transcriptome of these animals, we found that male and female mice had almost no common genes responsive to weekly prednisone. Instead, male skeletal muscle appeared to respond to weekly prednisone with genes implicated in hypertrophy and improved calcium handling, while females expressed genes underscoring a metabolic shift toward increased lipid utilization. We found evidence of a sex-specific GR DNA-binding pattern, consistent with weekly prednisone improving muscle performance through independent mechanisms in male and female mice.

## Results

### Weekly glucocorticoid treatment improves force in both sexes.

**Figure 1:**
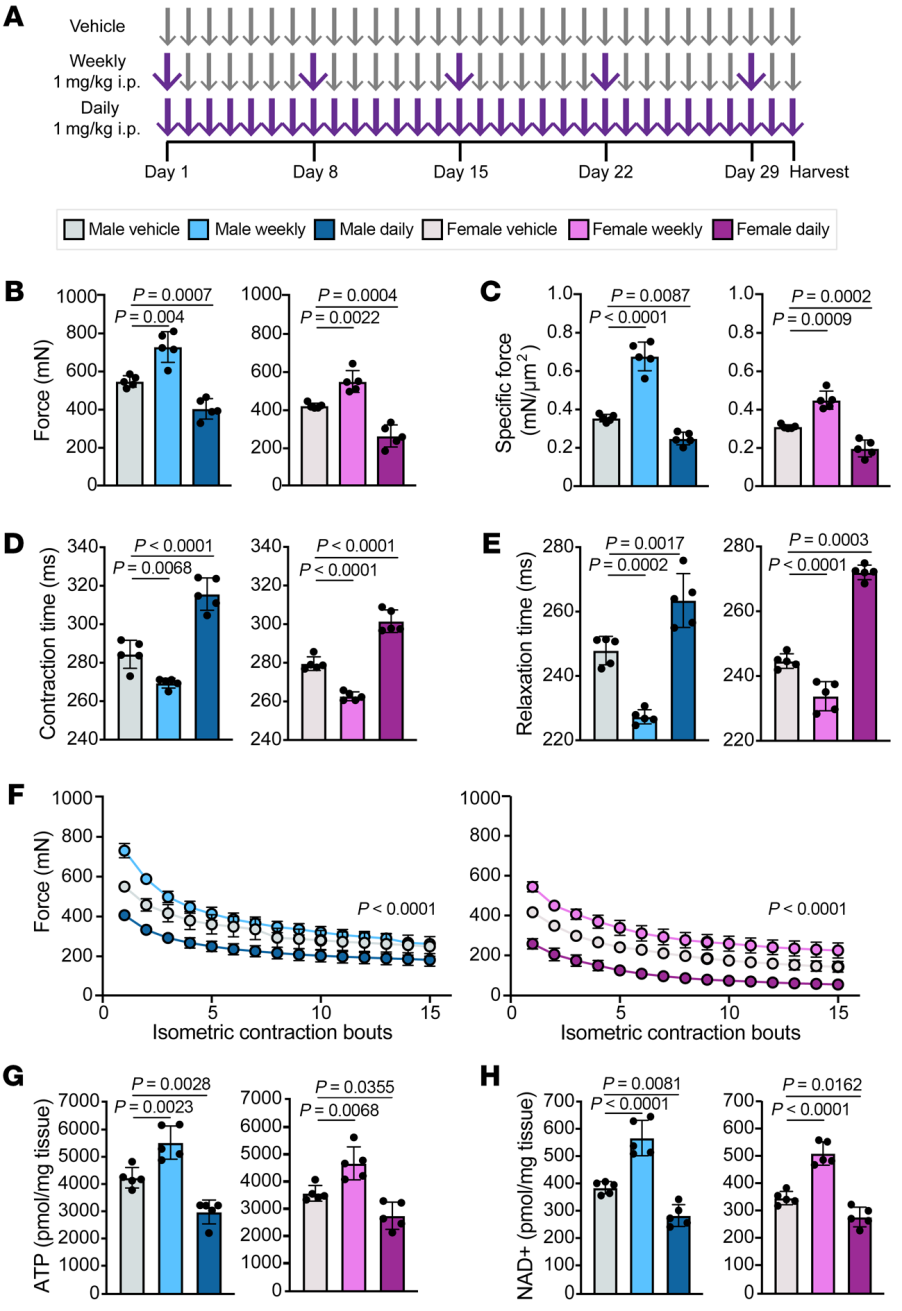
Weekly prednisone improved muscle performance in both male and female mice. (**A**) C57BL/6J mice were treated for 4 weeks with vehicle (DMSO) or weekly or daily prednisone, and then analyzed. (**B**–**F**) Weekly treated mice exhibited significantly increased maximal tetanic force (**B**) and specific force (**C**) and reduced contraction (**D**) and relaxation (**E**) time compared with vehicle-treated mice. Once-weekly prednisone–treated mice also exhibited increased response to repetitive force (**F**) compared with vehicle-treated mice. (**G** and **H**) Weekly treated mice had increased concentrations of ATP (**G**) and NAD+ (**H**) compared with vehicle-treated animals, while daily treated mice had significantly reduced concentrations. Data were analyzed with 1-way ANOVA (**B**–**E**, **G**, and **H**) or 2-way ANOVA (**F**).

Male and female 10-week-old C57BL/6J mice were treated with either weekly or daily injections of prednisone (1 mg/kg i.p.) for 4 weeks (Figure 1A). At the end of treatment, we analyzed muscle force, relaxation and contraction time, and response to repetitive force in the tibialis anterior muscles of each group. Daily prednisone treatment induced comparable atrophy in both male and female mice, leading to reduced maximal tetanic and specific force, increased contraction and relaxation time, and reduced force over consecutive isometric contraction bouts (Figure 1, B–F). In contrast, weekly prednisone treatment improved muscle performance in all metrics, suggesting that, unlike daily prednisone, weekly exposure to prednisone did not induce atrophy. Weekly prednisone–treated males and females had significantly more ATP and NAD+ per milligram of tissue compared with vehicle-treated animals (Figure 1, G and H). Daily treated animals had significantly less ATP and NAD+ than vehicle-treated animals, reflecting their atrophic state. Neither daily nor weekly prednisone treatment influenced blood glucose or serum insulin levels (Supplemental Figure 1, A and B; supplemental material available online with this article; https://doi.org/10.1172/JCI14982DS1). Chronic treatment with exogenous glucocorticoids is known to suppress endogenous glucocorticoids, and we indeed found that daily treatment resulted in a significant reduction in circulating corticosterone (Supplemental Figure 1C). Weekly prednisone did not suppress circulating corticosterone. To evaluate the effect of weekly prednisone on fully mature animals, we separately treated a cohort of 18-week-old mice for 4 weeks and found that they responded to weekly prednisone similarly (Supplemental Figure 2).

**Figure 2:**
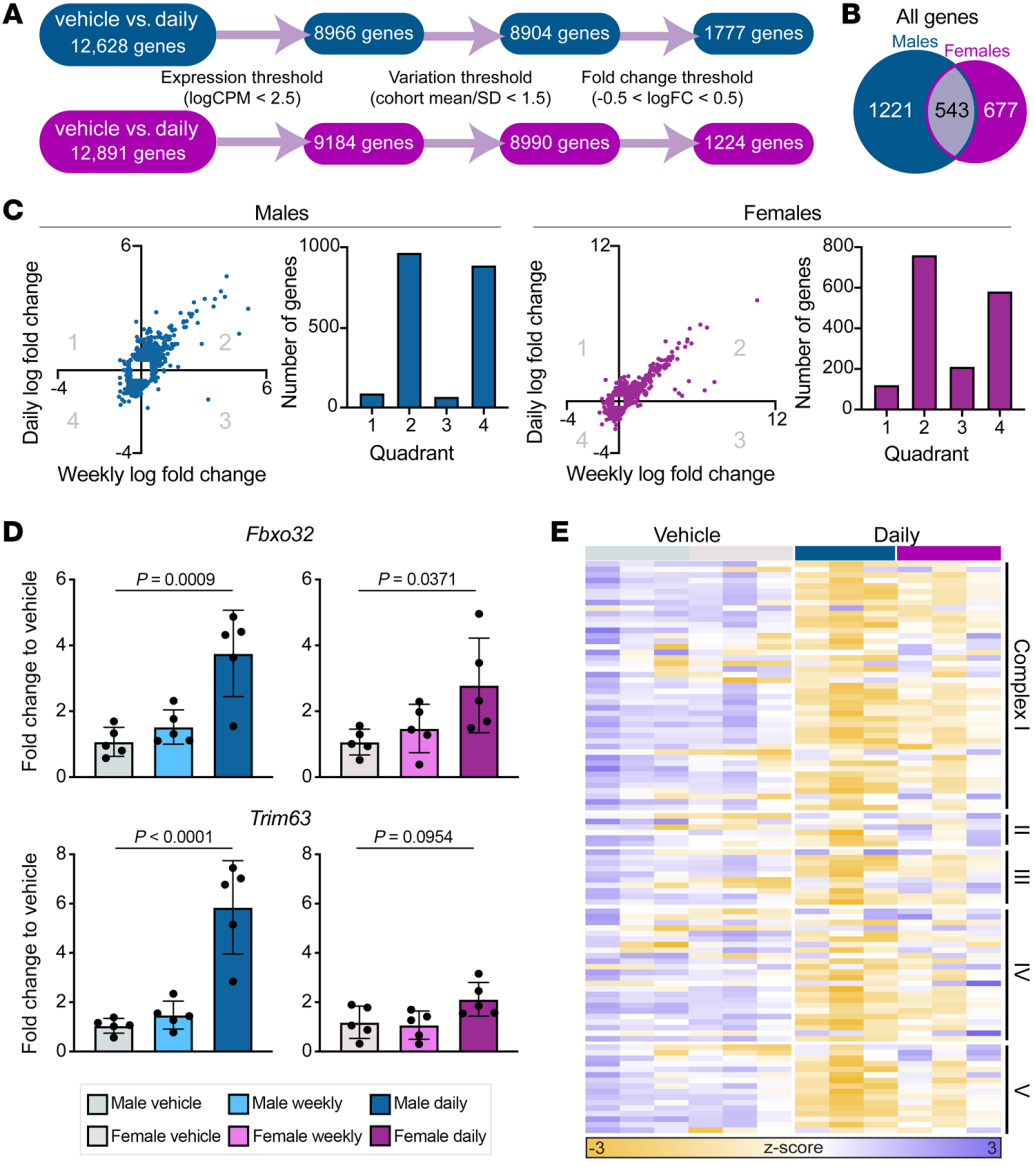
Daily and weekly prednisone treatment elicited similar transcriptional profiles with differential atrogene activation. (**A**) RNA sequencing analysis of daily prednisone–treated muscles (quadriceps) compared with vehicle-treated for both sexes; prednisone-responsive genes were identified as being above expression and fold-change thresholds and below a variation threshold; *n =* 3 animals per group. (**B**) Less than half of all prednisone-responsive genes were shared among daily prednisone–treated males and females. (**C**) The majority of prednisone-responsive genes above a log_2_(fold change) threshold had the same response to both daily and weekly treatment, i.e., increased (quadrant 2) or decreased (quadrant 4) expression. (**D**) Expression of atrogenes *Fbxo32* and *Trim63* was increased in daily treated muscle fibers compared with vehicle, as evaluated by qPCR and 1-way ANOVA, while weekly treated muscle fibers had no change in expression of these atrogenes. (**E**) Expression of genes encoding the mitochondrial respiratory chain was decreased in response to daily treatment in both sexes compared with vehicle treatment.

**Figure 3:**
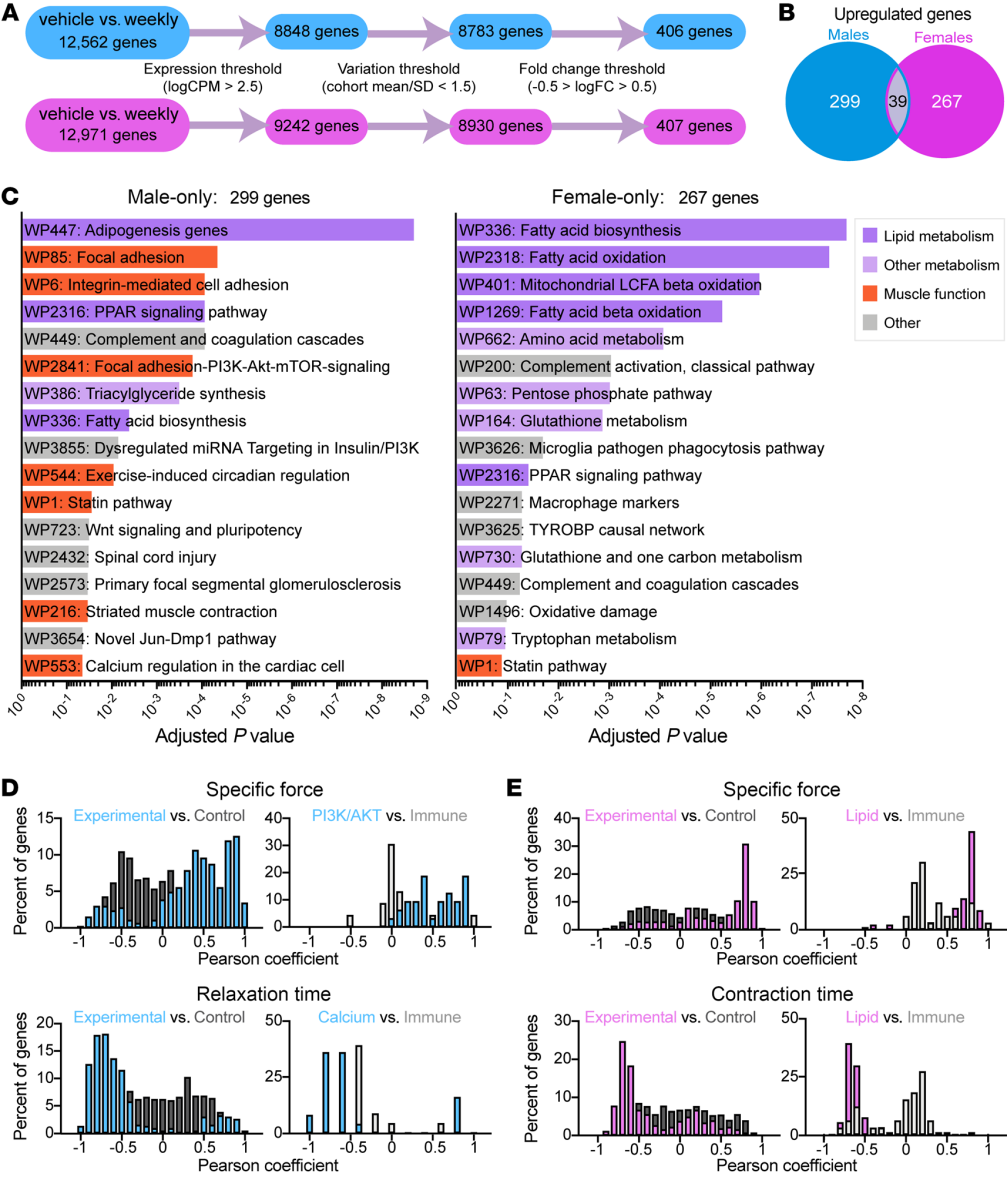
Weekly prednisone elicits distinct transcriptional response in male versus female muscle. (**A**) Transcriptome comparison of once-weekly prednisone–treated muscle to vehicle-treated for both sexes; prednisone-responsive genes were identified as being above expression and fold-change (FC) thresholds and below a variation threshold. *n =* 3 animals per group. (**B**) The majority of prednisone-responsive upregulated genes were unique to one sex. (**C**) Gene ontology analysis of male- and female-only upregulated genes shows differential pathway enrichment. (**D** and **E**) Pheno-RNA analysis of genes shows high correlation between gene expression and physiological response in males (**D**) and females (**E**). Genes responsive to weekly prednisone are more correlated with physiological response than a shuffled control group and genes in pathways of interest are more correlated with physiological response than prednisone-responsive genes involved in other cellular processes.

Glucocorticoids work primarily through their receptor, GR, a nuclear receptor transcription factor. We hypothesized that the differences we observed between daily and weekly prednisone–treated animals was the result of distinct transcriptional profiles. To investigate this, we performed RNA sequencing (RNA-Seq) on myofibers isolated from quadriceps muscle of male and female animals from each treatment group. After filtering for baseline expression, variability, and log_2_(fold change), in daily prednisone–treated muscle 1777 genes were changed in males and 1224 genes in females compared with vehicle-treated animals of each sex (Figure 2A). Less than half the genes were shared between the sexes (Figure 2B). In contrast, in weekly prednisone–treated muscles, 407 and 406 genes were changed in males and females, respectively (Figure 3A), and the majority were unique to one sex (Figure 3B). We asked how many genes had a divergent response to the 2 treatments, i.e., upregulated by one and downregulated by the other, and the vast majority of genes had the same directionality of transcriptional response after daily and weekly prednisone (quadrants 2 and 4, Figure 2C), suggesting that the transcriptional profiles were overall similar between daily and weekly treatment. We did find that daily versus weekly prednisone had distinct effects on the expression of the canonical mediators of glucocorticoid-induced atrophy. Expression of the genes encoding MuRF-1 (*Trim63*) and atrogin-1 (*Fbxo32*) was unchanged in weekly treated muscle compared to vehicle-treated, but these atrophy genes were upregulated in daily treated muscle (Figure 2D). In daily prednisone–treated muscle, we observed strong downregulation of the genes encoding the mitochondrial respiratory complex (Figure 2E). Taken together, these results suggest that daily and weekly prednisone treatment elicit some similar transcriptional effects in skeletal muscle, but daily prednisone exposure is distinguished by its atrophic gene expression pattern.

Having observed similarities in the transcriptional response between daily and weekly prednisone, we were curious whether administering prednisone twice a week would improve upon weekly prednisone or produce an effect more similar to daily exposure. We treated male and female mice with prednisone twice a week for 3 weeks (Supplemental Figure 3A). With twice-weekly treatment, both sexes had significantly more skeletal muscle NAD+ compared with vehicle, while only female muscle had significantly increased ATP (Supplemental Figure 3, B and C). Genes transcriptionally responsive to once-weekly prednisone were similarly responsive to twice-weekly (Supplemental Figure 3D-G), suggesting that while twice-weekly prednisone did not improve upon once-weekly, it also did not induce atrophy. This was further confirmed by quantitative PCR (qPCR) for *Fbxo32* and *Trim63*, which were not upregulated after twice-weekly prednisone treatment (Supplemental Figure 3H).

### Males and females have differing transcriptional responses to weekly glucocorticoid treatment.

Most of the transcriptional response to weekly prednisone treatment was through increased gene expression, suggesting weekly glucocorticoids can be considered primarily as an activating stimulus. Of those upregulated genes, the majority (87.2% in females, 88.4% in males) were unique to one sex (Figure 3B). Genes upregulated in weekly prednisone–treated females were predominantly involved in lipid metabolism pathways (dark purple, Figure 3C); however, genes upregulated in weekly treated males were involved in muscle function and growth pathways such as PI3K/AKT signaling and calcium regulation (orange, Figure 3C). To identify any associations between the transcriptional and physiological responses to weekly prednisone, we applied Pheno-RNA, a method that links genes to phenotypic outcomes (22). Compared with a group of control genes, genes responsive to weekly prednisone were more highly correlated with specific force and relaxation time in males (Figure 3D) and specific force and contraction time in females (Figure 3E). When we asked how well certain pathways correlated with physiological outcome, we found that PI3K/AKT signaling genes in males were more correlated with specific force than immune signaling genes, despite both pathways being significantly upregulated in response to weekly prednisone. Calcium-handling genes were more correlated with relaxation time than immune signaling genes in males. In females, lipid metabolism genes were more correlated with specific force and contraction time than immune signaling genes. Overall, we found that sex-specific transcriptional responses to weekly prednisone highly correlated with improvements in skeletal muscle performance.

**Figure 4:**
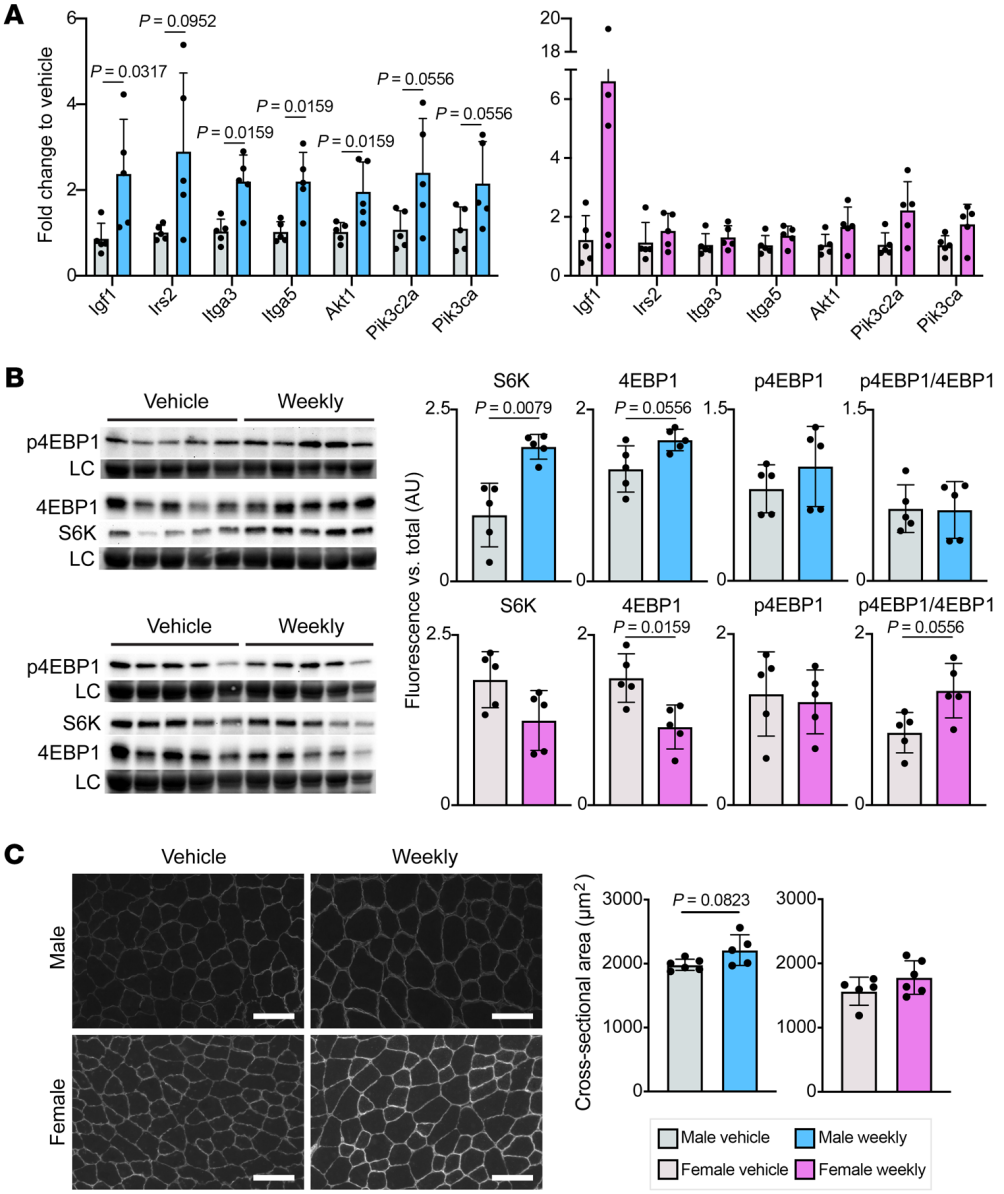
Weekly prednisone treatment activated the IGF1/PI3K pathway in males but not females. (**A**) Genes encoding IGF1/PI3K pathway members had increased expression in the muscle (quadriceps) of weekly treated male muscle but not female muscle by qPCR. (**B**) Total protein levels of mTOR targets S6K and 4EBP1 were increased in the gastrocnemius of weekly treated males but decreased in weekly treated females. LC, loading control. (**C**) Myofiber cross-sectional area in the tibialis anterior trended larger in weekly treated compared with vehicle-treated males but was unchanged in females. Scale bars: 100 μm. Data in **A**–**C** were analyzed with Mann-Whitney test.

The IGF1/PI3K pathway is upstream of mTOR and is known to regulate myofiber hypertrophy (23–25). Quantitative reverse transcription PCR (RT-PCR) confirmed upregulation of IGF1/PI3K pathway member genes in male but not female muscle (Figure 4A). mTOR is downstream of IGF1/PI3K, where it phosphorylates target proteins S6K and 4EBP1 (26). Total protein levels of S6K and 4EBP1 were significantly increased in weekly treated male muscle but were decreased in weekly prednisone–treated female muscle (Figure 4B). Levels of phosphorylated 4EBP1 were slightly increased in weekly prednisone–treated male muscles, resulting in a maintained p-4EBP1/4EBP1 ratio. Female muscle had an increase in the p-4EBP1/4EBP1 ratio, perhaps to compensate for a reduction in total 4EBP1. We measured myofiber cross-sectional area in the tibialis anterior muscle, as an indicator of hypertrophy; weekly prednisone–treated males trended toward increased cross-sectional area compared with vehicle-treated males, while there was no difference between female groups (Figure 4C).

**Figure 5:**
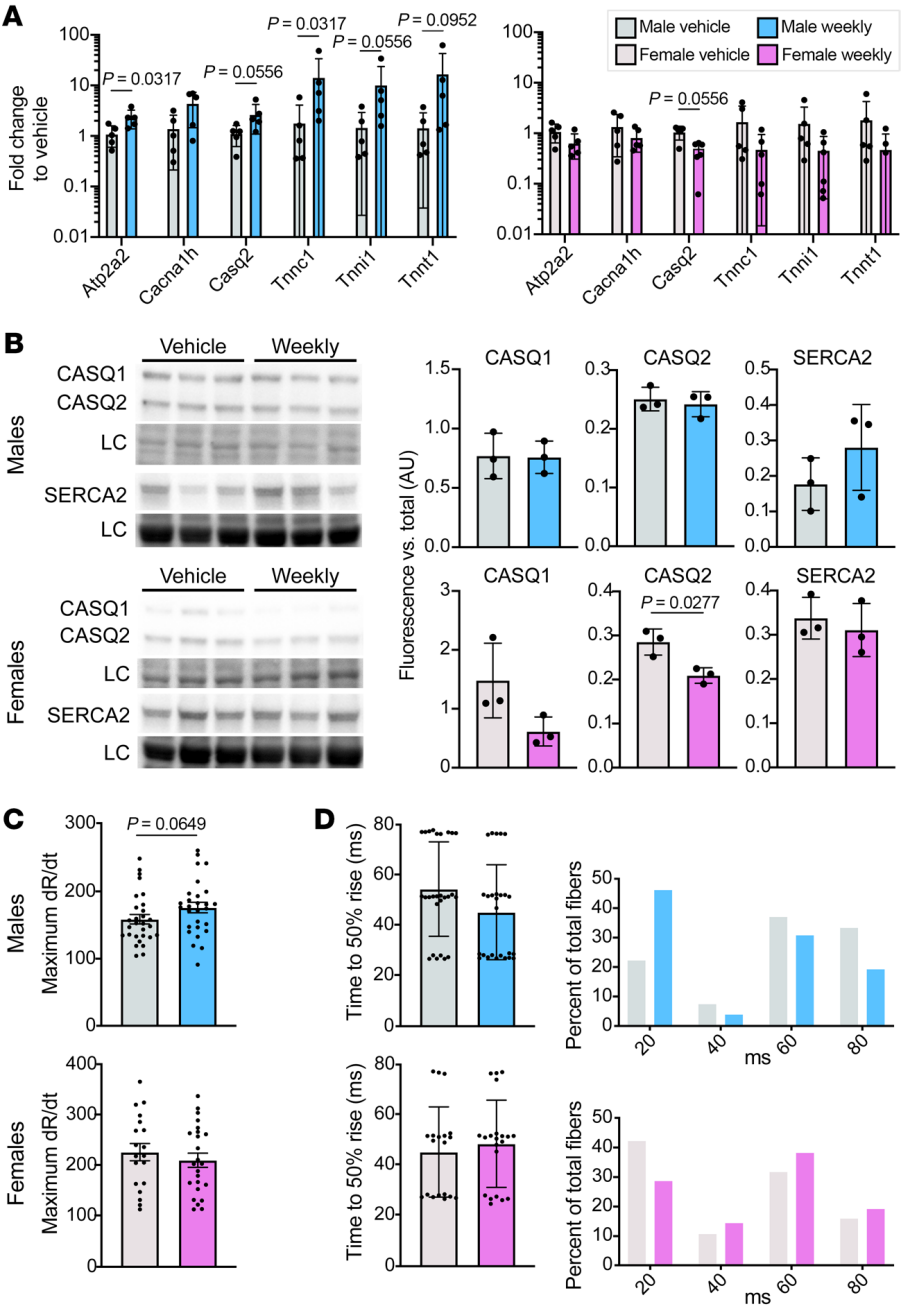
Weekly prednisone treatment improved calcium handling in males but not females. (**A**) Genes encoding calcium-handling proteins were upregulated in the quadriceps of weekly treated males but downregulated in weekly treated females by qPCR. (**B**) Protein levels of CASQ1, CASQ2, and SERCA2 were unchanged or increased in the gastrocnemius of weekly treated males, but decreased in weekly treated females compared with vehicle-treated animals. LC, loading control. (**C** and **D**) Calcium transient measurements in isolated flexor digitorum brevis fibers trended toward a faster maximum rate of calcium release (change in ratio/time, dR/dt) (**C**) and time to 50% rise (**D**) in weekly treated males and no change in weekly treated females compared to vehicle-treated animals. Data were analyzed with Mann-Whitney test (**A** and **C**) or Welch’s *t* test (**B**).

Gene ontology (GO) analysis of genes upregulated in male muscle also identified enrichment of genes involved in calcium handling (Figure 3C). Quantitative RT-PCR confirmed that genes encoding SERCA2 (*Atp2a2*), calsequestrin (*Casq2*), troponin (*Tnnc1*, *Tnni1*, and *Tnnt1*), and the Ca_v_3.2 channel (*Cacna1h*) were upregulated by weekly glucocorticoid treatment in males but were unchanged or downregulated in females (Figure 5A). Protein levels of calsequestrin isoforms 1 and 2 were not changed in response to weekly treatment in males, although SERCA2 did appear to be elevated (Figure 5B). In contrast, levels of both calsequestrin isoforms were reduced in weekly treated females. To evaluate how these gene expression and protein changes might impact calcium handling, we investigated calcium transients at 40 Hz in isolated flexor digitorum brevis (FDB) myofibers. Weekly treated males had an increased calcium release rate compared with vehicle-treated males, while females had no change (Figure 5C). Weekly treated males also had more fibers reach 50% rise with the first electrical stimulus compared with vehicle-treated males, while weekly treated females had fewer (Figure 5D). Overall, these data suggest that males had improved calcium handling and increased IGF1/PI3K pathway activity in response to weekly glucocorticoid treatment, while females did not.

### Weekly treatment changes lipid metabolism in females, resulting in increased endurance.

**Figure 6:**
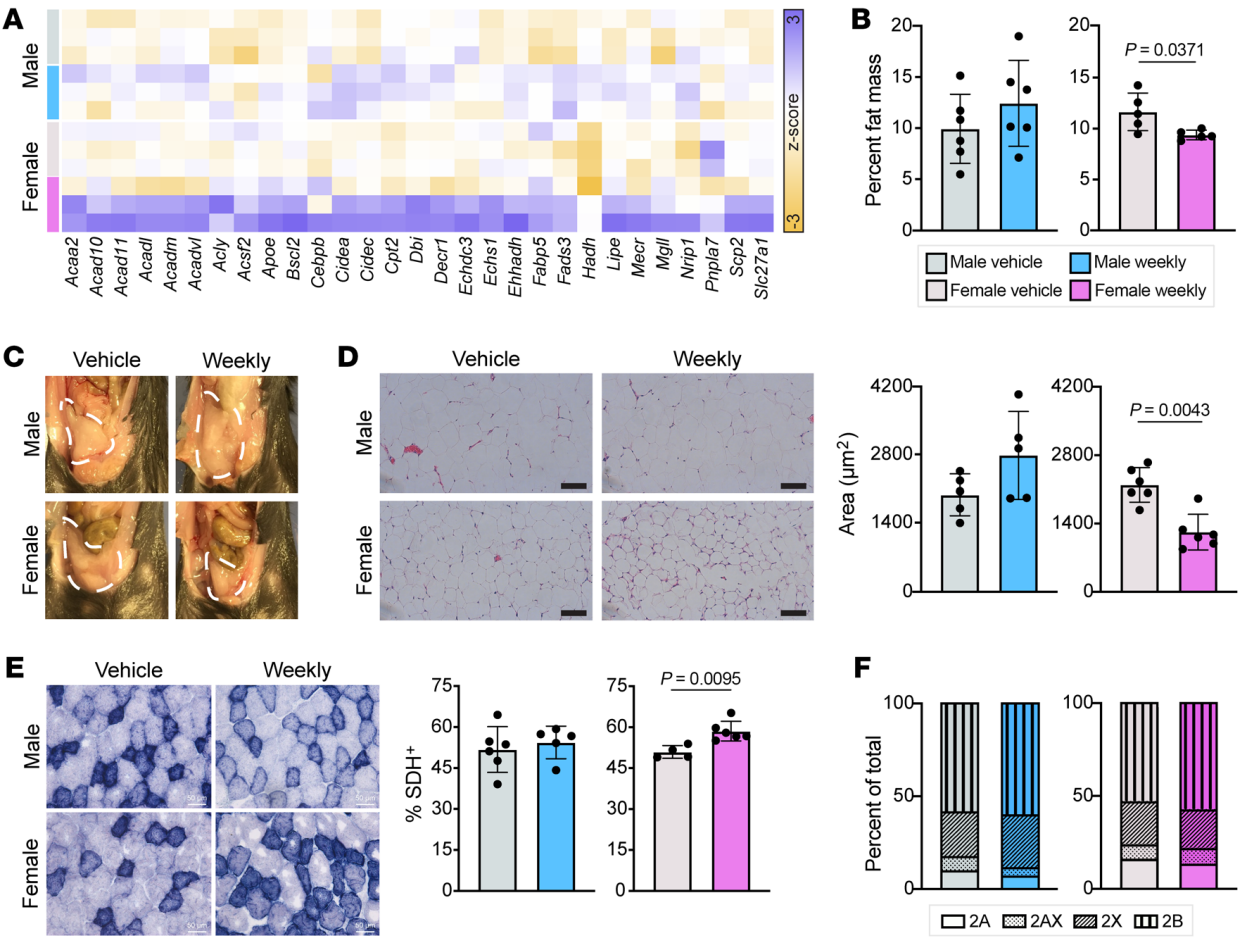
Weekly prednisone treatment improved lipid metabolism in females but not males. (**A**) Results from RNA sequencing analysis revealed increased expression of lipid metabolism–related genes in weekly prednisone–treated females compared with vehicle-treated females and weekly prednisone–treated males. (**B**) Body composition analysis after 4 weeks of treatment demonstrated reduced whole-body percentage fat mass in once-weekly prednisone–treated females, while comparably treated males had no change. (**C** and **D**) The visceral fat pad of weekly treated females was smaller than vehicle-treated females and weekly treated males (**C**) and the adipocytes in this fat pad had significantly reduced cross-sectional area (**D**). (**E**) Weekly treated females had an increased proportion of succinate dehydrogenase–positive (SDH^+^) fibers in the tibialis anterior compared with vehicle-treated females, while the males exhibited no change. Scale bars: 100 μm (**D**) and 50 μm (**E**). (**F**) Neither sex had changes in fiber type proportion following weekly treatment. Data in **B**, **D**, and **E** were analyzed with Mann-Whitney test.

GO analysis of genes upregulated in female muscle after weekly prednisone highlighted enrichment of lipid metabolism–related pathways (Figure 3C). Although expression of some lipid metabolism genes was slightly increased in weekly treated males, weekly treated females exhibited a unique and strong upregulation of genes involved in beta oxidation, lipid droplet formation, lipolysis, and fatty acid binding and transportation (Figure 6A). These expression shifts were confirmed by quantitative RT-PCR (Supplemental Figure 4). After 4 weeks of glucocorticoid treatment, weekly treated females had decreased whole-body percentage fat mass, while weekly treated males were unchanged compared with vehicle-treated animals (Figure 6B). These differences could be seen in the size of the visceral fat pad, which was much smaller in weekly treated females compared with vehicle-treated females and all males (Figure 6C). Interestingly, this difference appears to have been driven by adipocyte size; the cross-sectional area of adipocytes in the visceral fat pad was significantly reduced in weekly treated females (Figure 6D). Reflecting a change in metabolic state, an increased percentage of myofibers in the tibialis anterior muscles of weekly treated females were positive for succinate dehydrogenase enzymatic activity (Figure 6E), without any associated change in fiber type (Figure 6F).

**Figure 7:**
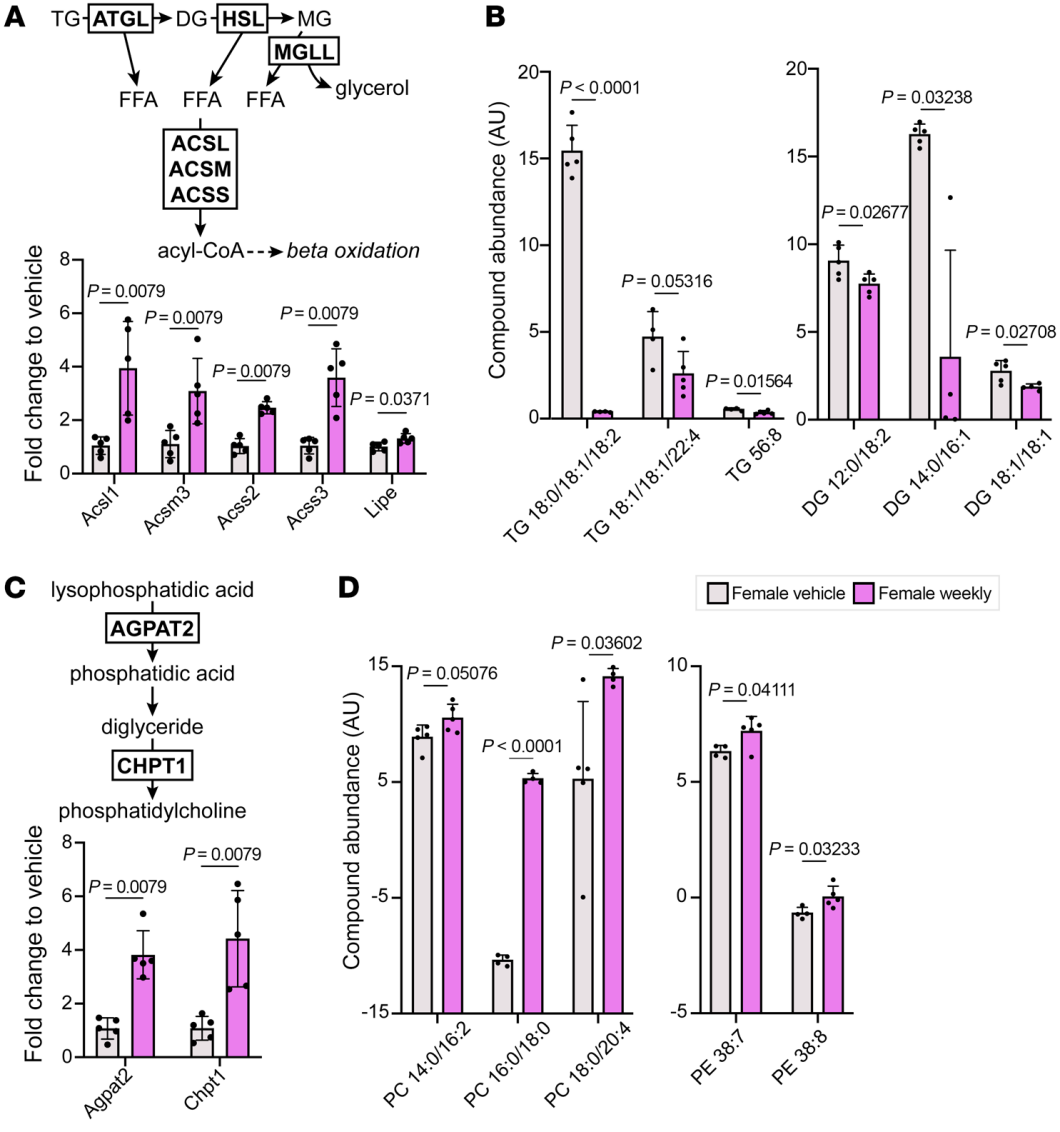
Weekly prednisone–treated females had increased glyceride catabolism and mitochondrial phospholipid abundance. (**A**) Muscles (quadriceps) from weekly prednisone–treated females had significantly increased expression of enzymes involved in triglyceride catabolism and acyl-CoA synthesis. (**B**) Females also had significantly reduced abundance of triglyceride and diglyceride species in the quadriceps. (**C**) Enzymes involved in phosphatidylcholine synthesis were significantly upregulated by weekly prednisone treatment. (**D**) Phosphatidylcholine (PC) and phosphatidylethanolamine (PE) abundance was increased in weekly prednisone–treated females compared with vehicle-treated females. Top 3 most abundant lipid compounds are presented as (number of carbon atoms):(number of double bonds); less abundant compounds are listed in Supplemental Table 4. Data were analyzed with Mann-Whitney test (**A** and **C**) or Welch’s *t* test (**B** and **D**).

To investigate the impact of weekly prednisone and associated changes in lipid metabolism on the skeletal muscle lipid profile, we performed untargeted lipidomics on whole quadriceps from vehicle- and weekly treated females. Overall, only 26 (4.8%) of 536 lipid compounds profiled were significantly different between vehicle- and weekly treated females, most of which were glycerides and phospholipids. Triglycerides are the predominant form of stored lipid in skeletal muscle and are funneled into beta oxidation through their catabolism into free fatty acids. Quantitative RT-PCR demonstrated upregulation of genes encoding enzymes involved in glyceride catabolism and acyl-CoA synthesis after weekly prednisone (Figure 7A). Di- and triglyceride species were significantly decreased in weekly treated females (Figure 7B and Supplemental Table 4), further suggesting that this pathway was activated by weekly prednisone. The phospholipids phosphatidylcholine (PC) and phosphatidylethanolamine (PE) are the main components of skeletal muscle mitochondrial membranes (27, 28). Genes encoding key enzymes involved in the synthesis of PC were upregulated by weekly prednisone (Figure 7C), and PC and PE species were significantly increased in weekly treated females (Figure 7D and Supplemental Table 4). These data indicate a subtle but significant shift in skeletal muscle lipid metabolism following weekly prednisone.

**Figure 8:**
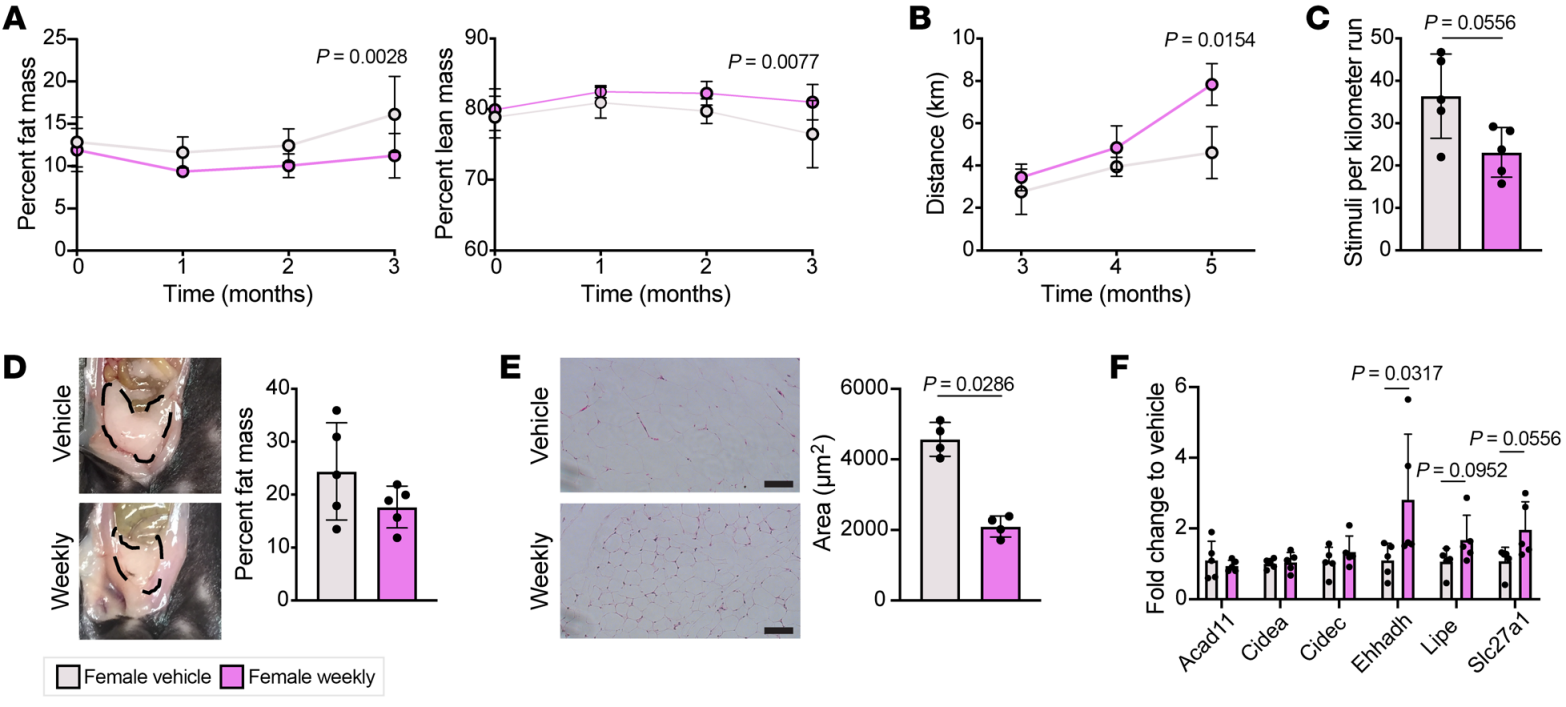
Long-term weekly prednisone treatment increased endurance in female mice. (**A**) Weekly prednisone–treated females had reduced whole-body percentage fat mass and increased percentage lean mass after 3 months of treatment. (**B**) Weekly prednisone–treated females showed increased distance to exhaustion in a treadmill run-to-exhaustion test compared with vehicle-treated females. (**C**) Weekly prednisone–treated females required fewer stimuli per kilometer run than vehicle-treated females. (**D**) After 9 months of treatment, weekly treated females had a slightly smaller visceral fat pad and moderately reduced percentage fat mass than vehicle-treated females. (**E**) Adipocytes in the visceral fat pad of weekly treated females had significantly reduced cross-sectional area compared with vehicle-treated females. Scale bars: 100 μm. (**F**) Some lipid metabolism genes continued to be upregulated in response to weekly prednisone. Data were analyzed with 2-way ANOVA (**A** and **B**) or Mann-Whitney test (**C**, **E**, and **F**).

We next tested whether exercise endurance, which is known to be influenced by lipid metabolism (29–31), was altered by this drug regimen. We treated a cohort of female mice with prednisone for 3 months and then assessed the running distance to exhaustion at 3 time points. The previously noted shift in body composition toward increased lean mass was maintained through 3 months of treatment (Figure 8A). Weekly treated females had a significantly increased distance to exhaustion after 6 months of weekly prednisone (Figure 8B), with fewer stimuli required per kilometer run (Figure 8C). Although their visceral fat pads were no longer significantly smaller than vehicle-treated females after 9 months of treatment (Figure 8D), weekly treated females still had significantly smaller adipocytes (Figure 8E). Some, but not all, of the lipid metabolism genes transcriptionally upregulated by 4 weeks of weekly prednisone were still upregulated in female muscle after 9 months of treatment (Figure 8F). Overall, we observed significant changes in the lipid metabolism of weekly treated females, including increases in expression of beta oxidation genes, triglyceride catabolism, and abundance of mitochondrial membrane phospholipids as well as decreased whole-body percentage fat mass, resulting in improved endurance.

### The sex-specific response to weekly prednisone is abrogated by sex steroid antagonism.

**Figure 9:**
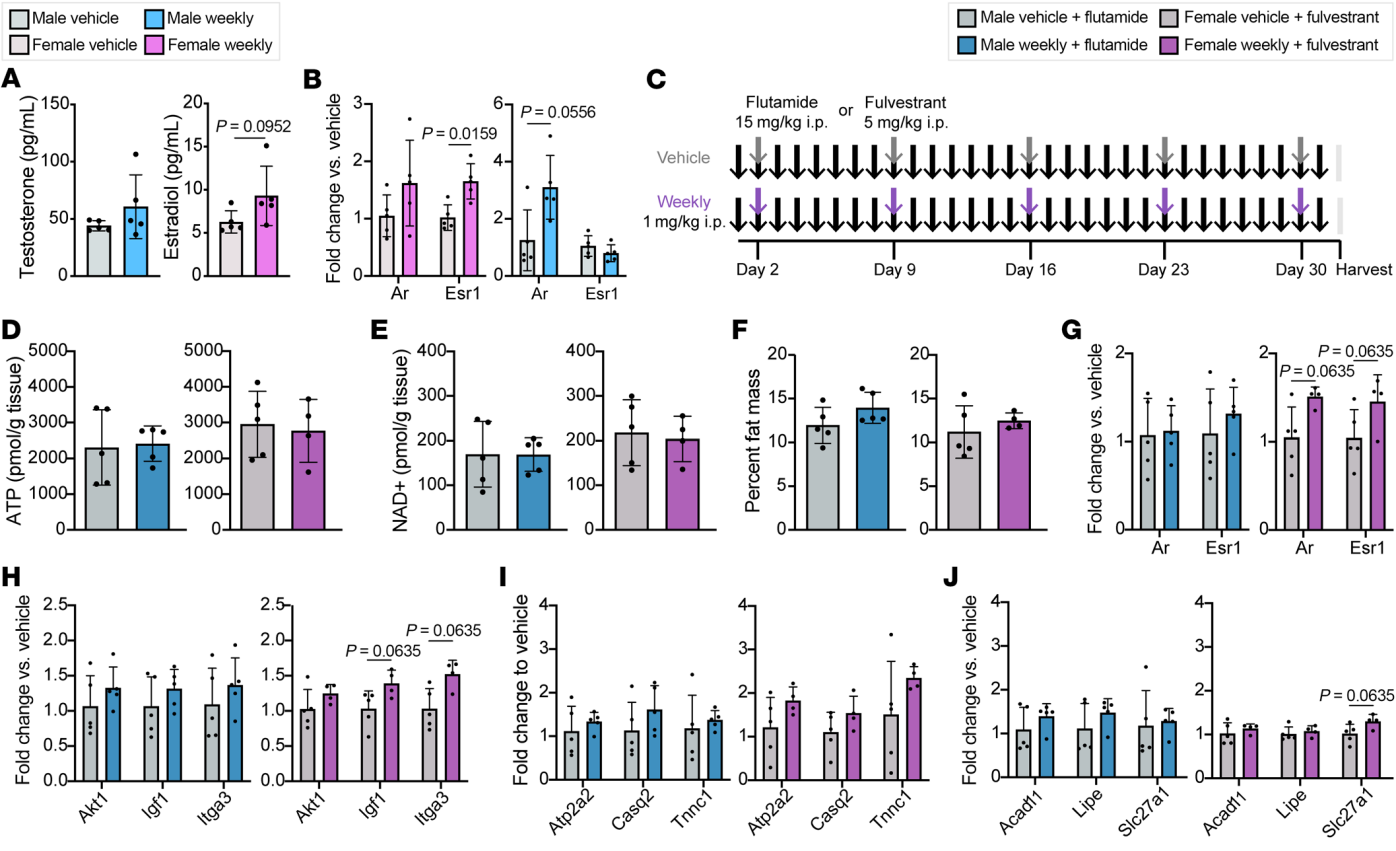
Sex steroid receptor antagonism attenuated the effects of weekly prednisone. (**A**) Circulating levels of testosterone and estrogen were increased in weekly prednisone–treated animals. (**B**) Expression of the gene encoding the androgen receptor (*Ar*) was increased in weekly prednisone–treated males, while expression of the gene encoding estrogen receptor α (*Esr1*) was increased in weekly treated females. (**C**) C57BL/6J mice were cotreated for 4 weeks with sex steroid inhibitors (males, flutamide; females, fulvestrant). Concomitantly, half of the cohort received weekly prednisone or vehicle. Arrows indicate i.p. injections. (**D** and **E**) Weekly prednisone–treated mice had no change in concentrations of ATP (**D**) and NAD+ (**E**) compared to vehicle-treated animals. (**F**) Weekly treated animals had no change in whole-body percentage fat mass when cotreated with sex steroid inhibitors. (**G**–**J**) After 4 weeks of weekly prednisone, males cotreated with flutamide had no change in gene expression profiles for sex steroid receptors (**G**), IGF1 pathway (**H**), calcium handling (**I**), or lipid metabolism (**J**). Females cotreated with fulvestrant had some increased sex steroid receptor (**G**), IGF1 pathway (**H**), and lipid metabolism (**J**) gene expression. Data were analyzed with Mann-Whitney test (**A**, **B**, and **D**–**J**).

Glucocorticoids regulate target gene expression through binding to GREs, a process moderated by many cofactors including the androgen receptor (AR) and estrogen receptor (ER). Both testosterone and estrogen were slightly increased in the serum in response to weekly treatment (Figure 9A) and expression of the genes encoding their receptors was sex-specifically responsive to weekly prednisone (Figure 9B). To investigate the role of these receptors in the response to weekly prednisone, we cotreated male mice with the AR competitive antagonist flutamide and female mice with the ER competitive antagonist fulvestrant in addition to vehicle or weekly prednisone starting at 10 weeks of age (Figure 9C). Four weeks of daily flutamide treatment significantly reduced body weight and prostate wet weight, while daily fulvestrant significantly reduced uterus wet weight but did not affect body weight (Supplemental Figure 5, A and B). When administered in conjunction with sex steroid antagonists, weekly prednisone failed to increased ATP and NAD+ in muscle (Figure 9, D and E) and did not reduce whole-body percentage fat mass or adipocyte cross-sectional area in female mice (Figure 9F and Supplemental Figure 5C). Weekly prednisone did not stimulate upregulation of *Ar* (Figure 9G), IGF1/PI3K pathway (Figure 9H), or calcium-handling genes (Figure 9I) in males cotreated with flutamide, suggesting that AR is required for the male-specific transcriptional response to weekly prednisone. Intriguingly, both *Ar* and *Esr1* were upregulated in females cotreated with fulvestrant (Figure 9G), as were the IGF/PI3K pathway members *Igf1* and *Itga3* (Figure 9H) and the lipid transporter *Slc27a1* (Figure 9J). Other lipid metabolism genes previously demonstrated to be responsive to weekly prednisone were not upregulated when females were cotreated with fulvestrant, suggesting that only part of the female-specific transcriptional response to weekly prednisone is modulated by ER.

### Weekly prednisone induces sex-specific chromatin changes.

**Figure 10:**
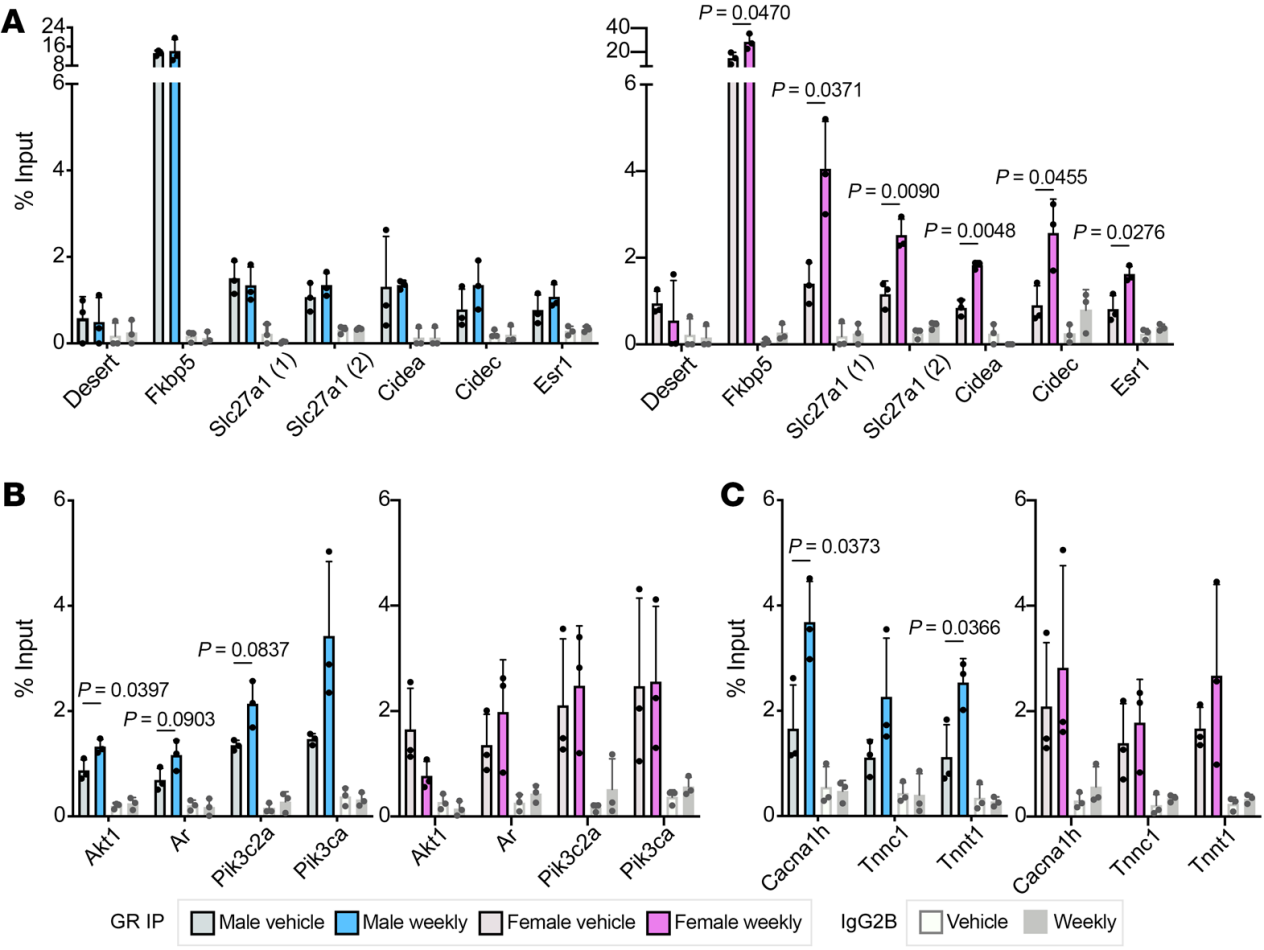
Sex-specific GR binding patterns in skeletal muscle treated with weekly prednisone. (**A**) ChIP-qPCR showed low occupancy of GR at a negative control locus on chromosome 5 (desert) and high occupancy at the canonical GRE near *Fkbp5* in both males and females. Putative GREs near lipid metabolism genes had increased occupancy of GR in weekly treated females compared with vehicle-treated females; males had no change in GR occupancy. (**B** and **C**) ChIP-qPCR showed increased occupancy at putative GREs near IGF1/PI3K pathway (**B**) and calcium-handling (**C**) genes in weekly treated males, while females had no change in binding with treatment. Data were analyzed with Welch’s *t* test (**A**–**C**).

To further investigate the sex-specific response to weekly prednisone, we performed chromatin immunoprecipitation (ChIP) for GR using vehicle- and weekly treated muscles. We investigated established or likely GREs in the following target genes: *Cidea*, *Cidec*, *Esr1*, *Pik3c2a*, *Pik3ca*, and *Tnnc1* (32–35). For the genes without previously identified GREs (*Akt1*, *Ar*, *Slc27a1*, and *Tnnt1),* we investigated binding at sequences matching the GR consensus motif, ACAnnnTGT, located within 10 kb of the transcriptional start site of the target gene. GREs near lipid metabolism genes had increased GR binding in weekly prednisone–treated females compared with vehicle, while weekly prednisone–treated males had no changes in GR occupancy (Figure 10A). For male muscle, GREs near IGF1/PI3K pathway and calcium-handling genes were enriched (Figure 10, B and C), including a well-characterized GRE known to modulate *Cacna1h* expression. These results demonstrate sex-specific GR binding profiles consistent with the sexually dimorphic transcriptional patterns.

**Figure 11:**
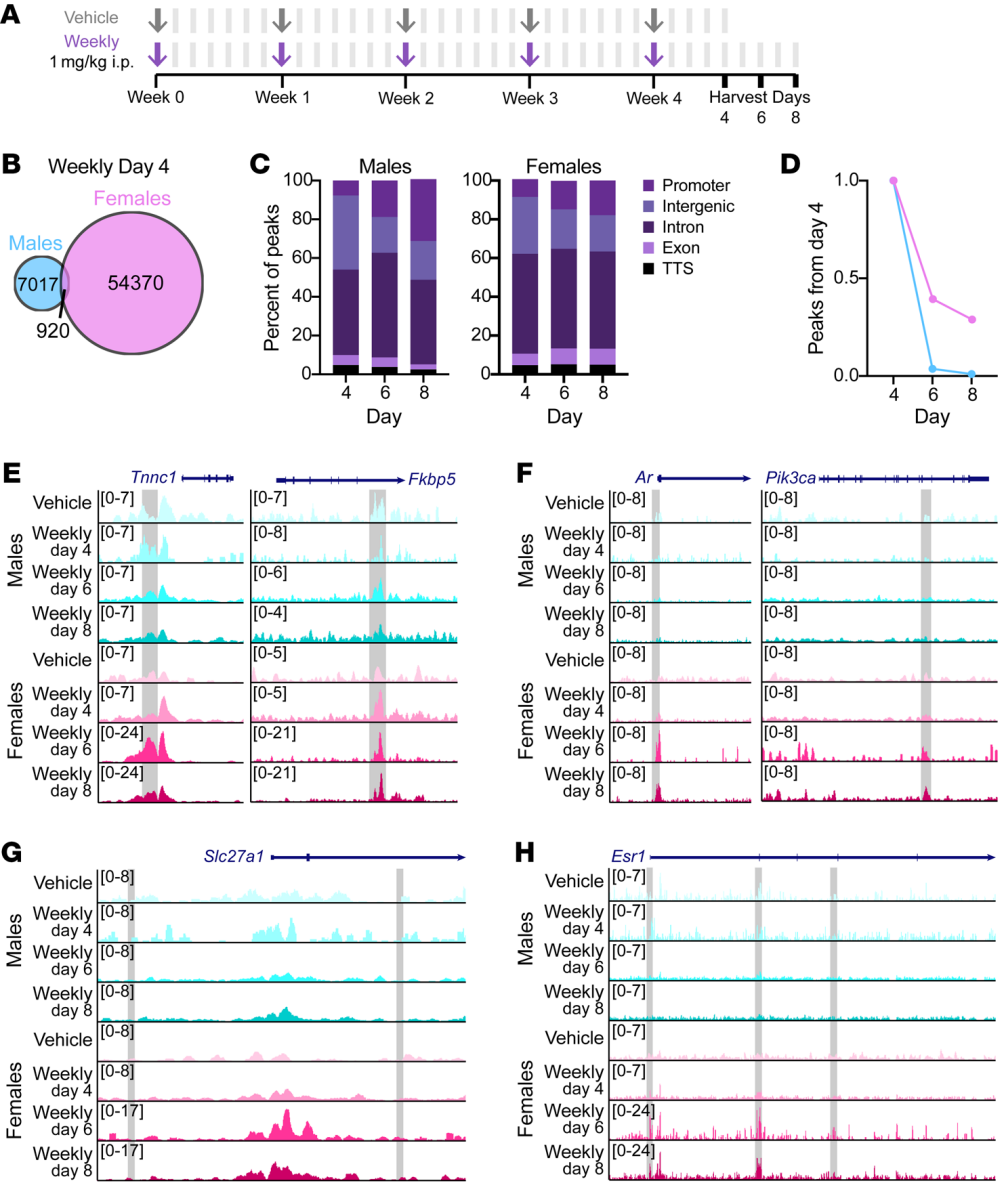
Skeletal muscle sex-specific enhancer landscape after weekly prednisone. (**A**) C57BL/6J mice were treated for 4 weeks with vehicle or weekly prednisone and then analyzed 4, 6, or 8 days after the final injection. Arrows indicate i.p. injections; bars indicate no injection. (**B** and **C**) Enhancers present 4 days after the final prednisone injection but not present in vehicle-treated myofibers were mostly unique to one sex (**B**) and predominantly intergenic or intronic (**C**). TTS, transcription termination site. (**D**) Percentage of enhancers present 4 days after final prednisone injection that were still present after 6 and 8 days. (**E**–**G**) ChIP-Seq tracks showing presence (**E**) or absence (**F** and **G**) of H3K27ac enrichment at putative GREs (gray boxes). (**H**) ChIP-Seq tracks showing female-specific enhancer peaks (gray boxes) near *Esr1*.

We investigated GR binding 2 days after the final weekly injection; our findings accordingly represent a relatively short-term response to prednisone, which has total body clearance in 24 to 36 hours in adult humans (36). To better understand how weekly prednisone affects chromatin architecture and how long those changes are maintained, we treated a cohort of mice for 4 weeks and then harvested quadriceps 4, 6, and 8 days after the final injection (Figure 11A). We performed ChIP-Seq for the histone modification H3K27ac on isolated myofibers to identify active enhancers (Supplemental Figure 6, A–C). We were particularly interested in enhancers present 4 days after the final weekly injection but absent in vehicle-treated samples, which we identified as prednisone-responsive enhancers (PREs). PREs were mostly intergenic or intronic and few were shared between males and females (Figure 11, B and C). We queried how many PREs remained at 6 or 8 days after the final injection. Although the vast majority of male PREs were lost by day 6, females maintained some PREs out to day 8 (Figure 11D). When we assigned the 500 top-ranked PREs to their nearest promoter, we found that males had PREs near genes implicated in muscle function and nuclear receptor signaling, while females had PREs near genes involved in IGF1/PI3K signaling (Supplemental Figure 6D). Those PREs maintained in females at day 8 were near genes involved in both lipid metabolism and IGF1/PI3K signaling (Supplemental Figure 6E). Similar to a previous investigation of dystrophic male muscle (20), the most significant transcription factor motifs enriched in long-lived PREs in both sexes were MEF2 family members (Supplemental Figure 6, F and G).

We then investigated H3K27ac enrichment at GREs with sex-specific binding 2 days after the final injection (Figure 10). Three of these GREs overlapped an enhancer that was present in all animals, including vehicle-treated ones, suggesting these enhancers had baseline activity that was further modulated by weekly prednisone (gray box, Figure 11E, and Supplemental Figure 6B). Some GREs with sex-specific GR binding in males at day 2 did not have H3K27ac enrichment at day 4 (Figure 11F), suggesting that these enhancers were transiently responsive to weekly prednisone. Transient enhancer occupancy was also observed at female-specific GREs (Figure 11G). Reflecting the overall longevity of the female response to weekly prednisone, we identified PREs near female-specific genes that were maintained out to day 8 (Figure 11H). Overall, these results suggest that male and female skeletal muscles undergo differential chromatin remodeling in response to weekly prednisone. While males had a relatively transient response to weekly prednisone that mostly disappeared by 6 days after the end of treatment, female skeletal muscle had more persistent chromatin marks that were evident for longer than a week after cessation of treatment. Intriguingly, these longer-lived enhancers annotate not only to genes involved in female-specific pathways such as lipid metabolism but also male-specific pathways such as IGF1/PI3K signaling.

## Discussion

### Weekly prednisone avoids triggering the atrophy pathways.

Weekly prednisone enhanced muscle performance, while daily prednisone triggered significant muscle atrophy. We observed significant improvements in specific force compared with vehicle-treated animals, which is similar to studies of anabolic steroids such as testosterone (37) and nandrolone (38, 39). Although there was considerable overlap of the transcriptional profiles from weekly and daily prednisone–treated muscle, only daily prednisone exposure produced atrogene upregulation and canonical glucocorticoid-induced atrophy (2). Atrogene upregulation was accompanied by profound repression of mitochondrial respiratory chain genes in daily prednisone–treated mice, and this is consistent with the known role of chronic glucocorticoid treatment in the suppression of mitochondrial oxidation (40). Whether reduction of mitochondrial oxidation genes results from the direct action of GR or an indirect side effect of atrophy is not known. Once-weekly prednisone did not elicit the same atrogene upregulation as daily steroid treatment, thus providing many of the positive adaptive changes without the adverse consequences.

### Sexual dimorphism in response to weekly prednisone.

The vast majority of genes responsive to weekly prednisone were upregulated uniquely in one sex. Skeletal muscle is known to be one of the most sexually dimorphic tissues in the body. Males and females exhibit major differences, ranging from fiber type composition to metabolism and gene expression (41–45). Studies investigating the molecular effects of glucocorticoids on skeletal muscle have often been evaluated in a single sex (2, 17, 46) or with results from both sexes analyzed in aggregate, missing the biological effects of sex-specific responses (3, 19, 20). Studies in liver (21) and more recently in skin (47) suggest males and females can have distinct responses to synthetic glucocorticoid treatment. Male muscle was characterized by increased IGF1/PI3K signaling activation, a pathway strongly implicated in muscle growth (25). We also identified a role for calcium handling in the male response to weekly prednisone. Glucocorticoids have been shown to increase store-operated calcium entry (48) as well as contractile force in isolated cardiomyocytes (49) and the mouse model of DMD (50), providing additional support for this mechanism of enhanced performance. Although we observed male-specific improvements in gene expression and calcium release rate, both sexes responded to weekly prednisone with significantly decreased muscle relaxation time. We suspect that these improvements in female skeletal muscle performance were the result of increased ATP, the cytoplasmic concentration of which is known to mediate the time course and amplitude of tetanic force (51). The benefits offered by prednisone-induced increased ATP content in female muscle might be only observable by whole-muscle mechanics and too subtle to be evaluated in single myofibers, and further study is required to investigate this important relationship. Although we did not directly identify the source of increased ATP, production of ATP in muscle has been shown to be stimulated by IGF1 signaling (52), mitochondrial calcium taken up from the sarcoplasmic reticulum (53–55), and mitochondrial membrane phospholipid composition (56), all of which were improved by weekly prednisone. However, further investigation is needed to clarify exactly how these pathways specifically modulate specific force independently of fiber size.

### Female-specific improvements in lipid metabolism and endurance.

Glucocorticoids are well-described regulators of white adipose tissue, modulating both adipocyte differentiation and fasting- and hormone-induced lipolysis (57, 58). Acute or short-term exposure to glucocorticoids in particular has been identified as a mediator of lipolysis from adipocytes (59–61). We observed reductions in adipocyte size, visceral fat pad size, and whole-body percentage fat mass in weekly treated females but not males. Skeletal muscle of weekly treated females exhibited strong upregulation of lipid metabolism–related genes previously reported as glucocorticoid responsive in adipose tissue (35) and as having sexually dimorphic expression in skeletal muscle (62–66). This baseline sexual dimorphism has been proposed as an explanation for why, although endurance exercise instigates a shift toward increased lipid utilization in both sexes, the effect is stronger in females, who have a lower respiratory exchange ratio and utilize 25% to 50% less glycogen than males, depending on exercise type and muscle group (41, 67–69). The lipid profile of weekly treated females exhibited improvements that mirror those observed in trained muscle. Both PC and PE are responsive to exercise intervention (70) and addition of PE to the skeletal muscle mitochondrial membrane has been shown to promote mitochondrial respiratory capacity (56). We also observed increased expression of PC synthesis enzymes, including *Chpt1*, which has been shown by skeletal muscle–specific knockout to be crucial for exercise tolerance (71). Sexual dimorphism in skeletal muscle lipid metabolism gene expression has been partly attributed to differences in fiber type, and female muscle tends to have a higher percentage of oxidative fibers than male muscle (43, 72–75). We did observe that female muscle had a slightly higher percentage of type 2A fibers than male muscle (15.2% and 9.8% of the tibialis anterior muscle, respectively), but we observed no significant changes in fiber composition in response to weekly prednisone. The relatively small contribution of type 2A fibers is likely insufficient to account for the functional improvements after weekly prednisone.

Sex hormones influence muscle size and contractility, metabolism, and response to exercise (45, 76–79). GRs and sex steroid receptors are members of the same superfamily, capable of cooperatively binding (80) or sharing cofactors (81), and we hypothesized that AR and ER were responsible for directing sex-specific GR binding. When AR was antagonized, male muscle failed to respond to weekly prednisone, suggesting that AR is the cofactor mediating the male response to weekly prednisone. Intriguingly, weekly prednisone administered to female mice treated with an ER antagonist resulted in upregulation of previously male-specific genes such as *Ar* and *Igf1*. We also found that female myofibers treated with weekly prednisone alone had H3K27ac enrichment at male-specific GREs. It is possible that the female-specific transcriptional profile is the result of direct activation of female-specific genes and repression of male-specific genes; further investigation is needed to clarify whether ER is the direct mediator of both of these gene regulatory programs.

### Implications for clinical use of glucocorticoids.

This work focused on the muscle response to weekly prednisone exposure between male and females, but glucocorticoids are primarily prescribed as antiinflammatory drugs. Whether there are sexually dimorphic differences in inflammation response is possible and may be more relevant in the disease or injury context. Other adverse effects of glucocorticoids may also exhibit sexually dimorphic responses. For example, central obesity is a common side effect of chronic glucocorticoid treatment (82) but our data show a reduction in fat mass and adipocyte size only in females. Osteoporosis is a highly significant adverse consequence of steroid use, and bone is also known to have strong hormonal influence and sexual dimorphism. Glucocorticoid-induced osteoporosis is in part driven by decreased osteoblast function through suppression of IGF1 (83), expression of which was significantly upregulated in weekly treated males but not females.

Overall, our data indicate that weekly glucocorticoid treatment is beneficial to skeletal muscle health and performance. Although male and female mice exhibit similar improvements in strength, the mechanisms by which they achieve this are very different, driven by distinct gene expression profiles associated with specific differences in chromatin occupancy of the GR. This work has considerable implications given the widespread nature of chronic glucocorticoid use.

## Methods

### Animals.

Male and female C57BL/6J mice were obtained from The Jackson Laboratory at 8 weeks of age. Mice were acclimatized for 2 weeks prior to treatment onset. Mice were randomly assigned to treatment cohorts. Unless otherwise specified, all animals were 10 weeks of age at treatment onset. For long-term experiments, mice were assigned to treatment group based on initial weight and percentage fat mass such that each treatment group began with the same mean body mass and mean percentage fat mass. Mice were housed in a specific pathogen–free facility and maintained on a 14-hour light/10-hour dark cycle, with the light cycle beginning at 6 am.

### Drug treatments.

Prednisone (P6254, Sigma-Aldrich) was resuspended in DMSO (D4540, Sigma-Aldrich) at 5 mg/mL each week. Daily treatment was 1 mg/kg body weight prednisone in 50 μL PBS given every day via i.p. injection. Vehicle treatment was the equivalent amount of DMSO proportional to body weight in 50 μL PBS. Weekly treatment was 1 day of prednisone treatment (1 mg/kg prednisone in 50 μL PBS) and 6 subsequent days of vehicle treatment (DMSO in 50 μL PBS). Unanesthetized mice were injected daily at 7 am with sterile BD Micro-Fine IV Insulin Syringes (14-829-1A, Thermo Fisher Scientific). Mice were weighed weekly and body weight was used for dosing calculations. Short-term (4-week) treatments consisted of 5 weekly prednisone injections total, with the final injection given 2 days prior to sacrifice. Long-term (38-week) treatments consisted of 39 weekly prednisone injections total, with the final injection given 2 days prior to sacrifice. AR activity was suppressed via the competitive antagonist flutamide (F9397, Sigma-Aldrich), which was administered at 15 mg/kg in 90% corn oil/10% ethanol. ER activity was suppressed via the competitive antagonist fulvestrant (I4409, Sigma-Aldrich), which was administered at 5 mg/kg in 95% corn oil/5% DMSO. Sex steroid receptor antagonists were administered daily as 100-μL i.p. injections.

### Muscle mechanics, treadmill, and body composition.

In situ tetanic force and contraction and relaxation time from the tibialis anterior muscle were evaluated at 9 am just prior to sacrifice using a Whole Mouse Test System with a 1-N dual-action level arm force transducer (300C-LR, Aurora Scientific). Mice were anesthetized with 3% isoflurane in 100% O_2_ and tetanic force and contraction and relaxation time were evaluated as previously described (46). Specific force was calculated using myofiber cross-sectional area, described below, after sacrifice. Mice were run on a treadmill (Exer3/6 without electrical stimulation grills, Columbus Instruments) at a 15° incline with a start speed of 1 m/min. Speed was increased by units of 3 m/min in 2-minute increments to a final speed of 15 m/min. The assay was terminated when mice stopped for longer than 15 seconds on the rest pad and could not be prodded to return to running. The number of times a mouse had to be prodded to run was recorded and reported as stimuli per kilometer run. Treadmill experiments were performed 2 days after the most recent weekly injection beginning at 8:30 am. Body composition was evaluated prior to and during treatment, occurring monthly 1 day after the most recent weekly injection at 1 pm. Percentage fat mass, percentage lean mass, and hydration ratio were determined in unanesthetized mice using MRI (EchoMRI). For all muscle mechanics, treadmill, and body composition assays, the operator was blinded to treatment group.

### FDB isolation and calcium indicator dye loading.

The FDB muscle was dissected from mouse hind-limb foot pads and incubated in DMEM (SH30022.01, HyClone) with 2 mg/mL BSA (SLBT0167, Sigma-Aldrich) and 40 mg/mL collagenase II (17101-015, Invitrogen) and was incubated at 37°C with 10% CO_2_ for 90 minutes (84). The FDB muscles were moved to Ringer’s solution (123 mM NaCl, 2 mM CaCl_2_, 5 mM KCl, pH 7.4) and triturated to isolate individual myofibers. MatTek 10-mm glass-bottom dishes were coated with 20 μg/mL laminin for 1 hour (23017-015, Gibco), after which the FDBs were adhered to the surface for 30 to 45 minutes at 37°C with 10% CO_2_. After 1 hour, the medium was exchanged with 300 μL of Ringer’s solution with 6 g/L glucose and 1% penicillin/streptomycin for overnight incubation at 37°C maintained in an incubator with 5% CO_2_. Leak-resistant Indo-1-AM (145, TEF Labs) cytosolic calcium indicator dye was resuspended in DMSO and 10% Pluronic. FDB muscles were loaded with 5 μM Indo-1 for 45 minutes in a 37°C incubator with 10% CO_2_.

### Calcium transient and sarcomere shortening measurements.

Isolated FDB muscles loaded with Indo-1 dye were imaged on a Nikon Diaphot inverted microscope equipped with a high-speed camera and photomultiplier tubes integrated with the FluoroDaq system (IonOptix) to measure cytosolic calcium transients and cell shortening as previously described (85). FDB muscles were paced with platinum electrodes designed for 35-mm MatTek dishes and connected to a high-voltage follow stimulator (701C, Aurora Scientific). Cells were paced at 40 Hz for 100 ms using a 0.2 ms pulse width at 18 to 20 V. A video sarcomere length system (900B, Aurora Scientific) was used to measure sarcomere spacing from bright-field images using a fast-Fourier transform. Aurora Scientific’s 950A calcium fluorescence analysis module was used to record the calcium transients and sarcomere length-shortening data and analyze the parameters of average transients. FDB isolation and calcium transient measurements were performed blinded to treatment group.

### HPLC.

Gastrocnemius muscle was flash-frozen immediately after sacrifice and then pulverized to powder using a mortar and pestle chilled on dry ice. Extraction and neutralization were performed as previously described (86). NAD+ and ATP were measured by HPLC as previously described (20) by an operator blinded to treatment group.

### Histology and immunofluorescence microscopy.

Cross-sectional area for white adipose tissue was evaluated from hematoxylin and eosin staining of 10-μm paraffin sections imaged with a Zeiss Axio Observer Z1 at 10× (LD A-Plan 10×/0.25 Ph1, Zeiss) for quantification and 20× (Plan-Apochromat 20×/0.8, Zeiss) for representative images. Adipocyte cross-sectional area was measured by hand in Fiji (87) from 3 images per animal. The percentage of fibers positive for succinate dehydrogenase was evaluated from 10-μm cryosections of tibialis anterior incubated with sodium succinate and nitroblue tetrazolium at 37°C for 90 minutes. Two whole sections from each animal were imaged as 10× tiles (EC Plan-Neofluar 20×/0.3, Zeiss) using a Zeiss Axio Imager M2; representative images were taken at 20× (Plan-Apochromat 20×/0.8, Zeiss). Fiber type was evaluated by immunofluorescence; briefly, 10-μm serial cryosections of tibialis anterior were fixed in acetone and then incubated overnight with primary antibodies against Myh7 (1:10), Myh2 (1:30), Myh4 (1:10), and Myh1 (1:30) (BA-F8, SC-71, BF-F3, and 6H1, respectively; Developmental Studies Hybridoma Bank) at 4°C. Sections were then incubated with secondary antibodies Alexa Fluor 488 goat anti–mouse IgG1 and Alexa Fluor 594 goat anti–mouse IgM (A21121 and A21044, Life Technologies). Two whole sections from each animal were imaged as 10× tiles with a Zeiss Axio Imager M2. The number of fibers stained each color was counted by hand in Fiji. Cross-sectional area for skeletal muscle was evaluated from 10-μm cryosections of tibialis anterior fixed in 4% paraformaldehyde (PFA) and incubated overnight with an antibody against dystrophin (1:1000; PA1-37587, Invitrogen) at 4°C. Sections were then incubated with Alexa Fluor 488 donkey anti–rabbit IgG (A32790, Life Technologies). Two whole sections from each animal were imaged as 10× tiles with a Zeiss Axios Imager M2, and then cross-sectional area was evaluated using the MATLAB program SMASH (88). All staining was performed, imaged, and analyzed with operator blinded to treatment.

### Serum collection and analysis.

At the end of treatment, mice were fasted for 4 hours and blood was collected by heparinized microhematocrit capillary tube (22-362-566, Thermo Fisher Scientific). Blood was allowed to clot for 30 minutes at room temperature and then centrifuged for 5 minutes at 12,000*g* and 4°C. Plasma was collected as supernatant and used to determine circulating insulin (NC9440604, Thermo Fisher Scientific), corticosterone (ADI-900-097, Enzo Life Sciences), estradiol (Calbiotech; conducted by the University of Virginia Center for Research in Reproduction), and testosterone (IBL; conducted by the University of Virginia Center for Research in Reproduction). Blood glucose was measured from a drop of blood collected from the tip of the tail with an AimStrip Plus glucometer (Germaine Laboratories). Insulin, corticosterone, estradiol, and testosterone levels were evaluated by an operator blinded to treatment group.

### Immunoblot analysis.

Relative protein abundance and phosphorylation were assessed by immunoblotting. Briefly, snap-frozen muscles were ground to powder using a dry ice–chilled mortar and pestle and then resuspended in 500 μL ice-cold 1× PBS with 1 mM CaCl_2_, 1 mM MgCl_2_ or 1× RIPA (10 mM Tris-HCl pH 8.0, 1 mM EDTA pH 8.0, 0.5 mM EGTA pH 8.0, 0.1% sodium deoxycholate, 140 mM NaCl), and EDTA-free protease inhibitor cocktail (11836170001, Sigma-Aldrich) and phosphatase inhibitor (4906837001, Sigma-Aldrich). Tissue was homogenized using the TissueLyzer II (Qiagen) for 6 minutes at 30 Hz, resting every 2 minutes. Samples homogenized in RIPA were then incubated with 1% Triton X-100 (9002-93-1, Sigma-Aldrich) and 0.1% SDS (L3771-500G, Sigma-Aldrich) for 1 hour at 4°C. Samples were centrifuged to pellet insoluble material and then supernatant total protein was quantified using Bradford reagent (5000205, Bio-Rad). Protein was separated using 4%–15% gradient or 7.5% TGX gels (4561086 and 4561026, Bio-Rad) and then transferred to PVDF membranes (1620177, Bio-Rad) for 3 hours at 300 mA and 4°C. Blots were blocked for 90 minutes at room temperature in StartingBlock T20 (37543, Thermo Fisher Scientific) and incubated with primary antibody diluted in T20 overnight at 4°C. Blots were washed 4 times in 1× TBS with 0.1% Tween 20 (TBST) (BP337-500, Thermo Fisher Scientific), incubated for 1 hour at room temperature with secondary antibody conjugated to HRP, and washed again in TBST. Blots were imaged with SuperSignal West Pico Chemiluminescent Substrate (34080, Thermo Fisher Scientific) using an iBright CL1000 (Thermo Fisher Scientific). Total protein was assessed using the Pierce Reversible Protein Stain Kit (24585, Thermo Fisher Scientific). Densitometry was performed using Fiji and protein was normalized either to the total protein band at 42 kDa or as the phosphorylated-to–total protein ratio.

Antibodies used for immunoblots are listed in Supplemental Table 1.

### RNA-Seq analysis and quantitative RT-PCR.

Gene expression analysis was performed on RNA extracted from myofibers isolated from quadriceps with TRIzol (15596018, Life Technologies). Briefly, directly following harvest, the right quadriceps was cut in half and then sliced following the natural myofiber orientation before being incubated in collagenase II for 1 hour at 37°C . The muscle was manually dissociated and then filtered through a 40-μm cell strainer (22-363-547, Thermo Fisher Scientific). The unfiltered fraction was collected and processed with TRIzol. RNA was then isolated using the PureLink RNA Mini Kit (Thermo Fisher Scientific) per the manufacturer’s instructions and resuspended in 30 μL of RNase-free water. Quality controls, sequencing, alignment to mm10, and read count quantification and counts per million were performed as described previously (46). Raw reads are available in NCBI’s Gene Expression Omnibus database (GEO GSE168964). Heatmaps were visualized from *z* scores with medians in RStudio (89). GO analysis was conducted via the Wiki Pathways Mouse 2019 module in Enrichr (90, 91). Results from RNA-Seq were validated by quantitative RT-PCR with primers listed in Supplemental Table 2 using iTaq Universal SYBR Green Supermix (1725124, Bio-Rad). RT-PCR results for each gene are reported as fold change normalized to the average of the vehicle-treated samples. Correlation between gene expression and phenotypic data was assessed using Pheno-RNA (22). Genes with log counts per million greater than 2.5 and absolute log fold change greater than 0.5 (721 females, 470 males) were used for the analysis along with specific force, relaxation time, and contraction time. Pearson’s correlation coefficient was calculated for each gene and phenotype in 3 animals per sex per treatment group. A control group was generated by shuffling counts per million between samples as described in the original publication (22).

### ChIP-qPCR analysis.

ChIP was performed on chromatin isolated from snap-frozen whole left quadriceps. Muscle was ground to powder on dry ice and then homogenized 6 times in 2 mM disuccinimidyl glutarate (20593, Thermo Fisher Scientific) in a dounce homogenizer on ice. The muscle homogenate was filtered through first a 100-μm and then 40-μm cell strainer (22-363-549 and 22-363-547, Thermo Fisher Scientific) and the filtrate was incubated for 20 minutes at room temperature. The cells were pelleted and then resuspended in 1% PFA for a 5-minute fixation at room temperature. Pellets were resuspended in Fast IP Buffer (5 mM EDTA pH 7.5, 50 mM Tris pH 7.5, 0.05% NP-40, 150 mM NaCl) and lysed by passing through a sterile BD Micro-Fine IV Insulin Syringe (14-829-1A, Thermo Fisher Scientific) twice. Chromatin was sheared via Bioruptor Ultrasonicator (Diagenode), 6 cycles, 30 seconds on/off, and then spun down to remove impurities. The supernatant was split in half and incubated with 6 μg of either anti-GR antibody (sc-393232, Santa Cruz Biotechnology) or an IgG2B isotype control (MAB004, R&D Systems) overnight at 4°C, with 5% reserved as input. Protein A/G Magnetic Beads (88803, Thermo Fisher Scientific) were preblocked with 0.5% BSA in PBS overnight at 4°C. After overnight incubation, the antibody-chromatin mix was added to the magnetic beads and incubated for 6 hours at 4°C. Beads were washed 6 times with ice-cold Fast IP Buffer, twice with ice-cold TE pH 8.0, and then eluted at room temperature (105 mM sodium bicarbonate, 1.05% SDS). Following an overnight incubation at 65°C to reverse crosslinks, DNA was purified with a QIAquick PCR Purification Kit (28106, Qiagen) and eluted with 30 μL EB. GR enrichment was determined as percentage input by quantitative PCR. Positive and negative control loci as well as putative GREs identified are listed in Supplemental Table 3.

### ChIP-Seq analysis.

ChIP was performed on 2 biological replicates per treatment group on isolated myofibers from the left quadriceps. Briefly, myofibers were isolated as described above and then snap-frozen after filtering. Snap-frozen myofibers were ground to powder on dry ice and then fixed in 1% PFA for 5 minutes at room temperature. Chromatin was isolated and sheared as described above before being incubated with 5 μg of anti-H3K27ac antibody (39133, Active Motif) overnight at 4°C, with 5% reserved as input. Immunoprecipitation was performed as described above with a high-salt wash buffer (50 mM Tris pH 7.5, 500 mM NaCl, 0.1% SDS, 1% Triton X-100). Quality controls, library preparation, and sequencing were performed as described previously (20). Reads were trimmed to remove TruSeq adaptor sequences with homerTools (92) and then aligned to mm10 with Bowtie2 (93, 94). Peaks were called with HOMER using command findPeaks with parameters -style histone -F 2 with input tag directories used as controls. Peaks unique to weekly prednisone–treated skeletal muscle were identified by comparing vehicle- and weekly treated peak files using mergePeaks with parameter -d given. Significantly enriched motifs were identified using findMotifsGenome with parameters -size given -mask. Raw reads and peak files are available in the GEO database (GSE188302). Regions called as peaks or not peaks were validated by qPCR using primers listed in Supplemental Table 3.

### LC-MS/MS analysis.

Untargeted lipidomics was performed on muscle from female mice treated for 4 weeks with vehicle or weekly prednisone. Quadriceps muscle were snap-frozen and then ground to powder with dry ice–chilled mortar and pestle. Frozen samples were sent on dry ice to the Mass Spectrometry Core at the University of Illinois Chicago, where LC-MS/MS and data analysis were performed. Briefly, 10 μL of SPLASH Lipidomix was added as an internal standard prior to extraction. The lipid extracts were dried and resuspended in 100 μL methanol/chloroform (9:1, v/v) prior to analysis. MS analysis of the crude lipid extracts was performed using an Agilent 6545 Q-TOF LC–MS system controlled by the Agilent Mass Hunter acquisition software. The mass spectrometer was operated in 2 GHz extended dynamic range mode, employing precursor ion analysis for relative quantification experiments in positive ion mode. In positive ion mode, internal reference mass calibration ions *m*/*z* 121.0509 and 922.0098 were used. Mobile phase A was H_2_O/methanol (90:10, v/v) with 10 mM ammonium acetate and 0.5 mM ammonium fluoride. Mobile phase B was isopropanol/methanol/acetonitrile (5:3:2, v/v/v) with 10 mM ammonium acetate and 0.5 mM ammonium fluoride. The resuspended lipid extracts (2 μL) were loaded onto a 2.1 × 100 mm Agilent Poroshell C18, 2.7 μm column (Agilent Technologies Inc.) and separation performed using an Agilent 1290 UPLC system with the following gradient: 70% B at 0–1 minute, 86% B at 3.5–10 minutes, 100% B at 11–17 minutes, at a flow rate of 400 μL/ min. A postcolumn equilibration time of 5 minutes was used for all runs. Source parameters were as follows: gas temp 200°C, drying gas 11 L/min, nebulizer 35 psi, sheath gas temp 350°C, sheath gas ﬂow 12 L/min, VCap 3000 V, and fragmentor 145 V. Data were collected for relative quantification using a scan speed of 4 MS spectra per second. Pooled samples were prepared by combining 5 μL of each of the samples and an iterative MS/MS workflow was performed in the Mass Hunter acquisition software across 6 injections of the pooled samples with a scan speed of 10 MS and 3 MS/MS spectra per second.

### Lipidomics data analysis.

Raw LC-MS/MS data were used to create a fragmentation-based (MS/MS) library containing *m*/*z* precursors and retention times for all MS/MS-identified lipids using Lipid Annotator software (Agilent Technologies Inc.) and the following settings: lipid species for positive ([M + H]^+^, [M + Na]^+^, [M + NH_4_]^+^, [M + H – H_2_O]^+^, [M + Na – H_2_O]^+^, [M + NH_4_ – H_2_O]^+^), *Q* score of 60 or greater, and mass deviation of 10 ppm or less. Raw LC-MS data files were processed using the Profinder software (vB.10.00, Agilent Technologies Inc.). Here, molecular features were extracted for peaks with 5000 or greater counts in the positive mode ([M + H]^+^, [M + Na]^+^, [M + NH_4_]^+^, [M + H – H_2_O]^+^, [M + Na – H_2_O]^+^, [M + NH_4_ – H_2_O]^+^) using an isotope model of common organic molecules (no halogens). The resulting compound list was further filtered for those having an absolute height of 10,000 counts or greater, quality score of 60 or higher, and compounds having 2 or more isotopes present. Additionally, retention time for each compound was aligned to ±0.1 minute using a mass accuracy window of 5.0 ppm or less and peaks integrated using the Agile integrator in the Profinder software. Each of the integrated peaks was manually reviewed for retention time and fragmentation matching. The processed data file was then exported as a .cef and imported into the Mass Profiler Professional software (v15.1, Agilent Technologies Inc.) where each data set was analyzed separately. All compound abundance values were baseline corrected to the median abundance and normalized by the closest lipid standard. Compounds that were not present in all biological replicates of either treatment group were further filtered. Outliers were identified by Dixon’s *Q* test and outliers falling outside the 95% confidence interval were removed. Compounds of interest were identified by Welch’s *t* test as having a *P* value of less than or equal to 0.05 and are listed in Supplemental Table 4.

### Statistics.

Statistical analyses were performed with Prism (GraphPad). When comparing 3 groups, analyses were performed as either 1-way ANOVA (for treatment as the only variable) or 2-way ANOVA (for time and treatment as variables). When comparing 2 groups, the Mann-Whitney-Wilcoxon test was used. For analyses with small sample numbers, a Welch’s *t* test with was used. For ANOVA and *t* test analyses, a *P* value of less than or equal to 0.05 was considered significant, although all *P* values less than 0.1 are reported in figures. Dot plots and marked line plots depict mean ± SD.

### Study approval.

The study was conducted with the approval of the Institutional Animal Care and Use Committee at Northwestern University.

## Author contributions

IMS conceived experiments, carried out analyses, and drafted the manuscript. PGP, GT, and MH assisted in the animal experiments. MQ carried out muscle force analyses and provided editorial comments on the manuscript. DYB and IT assisted with measurements of calcium transients, and DYB provided critical edits to the manuscript. EMM is responsible for data integrity, study design, analyses, and editing the manuscript.

## Supplementary Material

Supplemental data

## Version 1 02/10/2022

In-Press Preview

## Version 2 03/15/2022

Electronic publication
