# Supplementary material for: Intermittent glucocorticoid treatment enhances skeletal muscle performance through sexually dimorphic mechanisms
Source: J Clin Invest. 2022 Mar 15;132(6):e149828. doi: 10.1172/JCI149828 (PMC8920338; doi:10.1172/JCI149828)
Supplement: Supplemental data [file jci-132-149828-s177.pdf]

SUPPLEMENTAL INFORMATION

Supplemental Table 1. Antibodies used for immunoblot

Antibody	Dilution	Catalog #	Source
4EBP1	1:1,000	A300-501A	Bethyl
phospho-4EBP1	1:1,000	2855	Cell Signaling Technology
S6K	1:1,000	A300-510A	Bethyl
CASQ1/2	1:1,000	ab3516	Abcam
SERCA2	1:10,000	MA3-919	Thermo Fisher
Goat anti-mouse HRP	1:2,500	115-035-003	Jackson ImmunoResearch Laboratories
Goat anti-rabbit HRP	1:2,500	111-035-003	Jackson ImmunoResearch Laboratories

Supplemental Table 2. qPCR primers

Gene	Forward Primer (5' to 3')	Reverse Primer (5' to 3')
Acad11	TAACGGCAAGAAGTGGTGGA	TGTGCTGTCTGTGTCTGGAT
Acsl1	GACTTGTTGAAACTTGGGAA	CATCTATCTGCGACCTGAAA
Acsm3	CCCAGCAGTAGATGCCGTG	TTCGTCGTGTTTTGTGTCCA
Acss2	GCAACTACAAACATCTGCTACA	ATCTTGGTGGTCTCCCCTG
Acss3	AAGTCTTCCGAGTTCCCGTT	CCTGGTGGAGGTGTTTTGG
Agpat2	CATCATCCCCGTGGTGTA	GAAATCTGTAGAAAGGTGGC
Akt1	GCCTGCCCTTCTACAACCA	CATACACATCCTGCCACACG
Ar	ACTATTACTTTCCACCCCA	CAGAGTCATCCCTGCTTC
Atp2b2	AATGCCCGCTGTTTTGCT	ATCTGCCAGGACCATCTCA
Cacna1h	CCCATCAACCCCACCAT	AGCATAGATAAAAAACAGGAG
Casq2	TGCGGAGAAGAGTGACCC	AGCAACAAGCAGTGGAAAGT
Chpt1	GCTCATTGGCAGACTTACG	GTCCCACATTGTTGCTCCT
Cidea	TTCCTCGGCTGTCTCAA	CAGATTCCTTAACACGGC
Cidec	ATCGGAAGGTTCGCAAAGG	CCAGCACCAGGGAGAAGG
Ehhadh	CTTGGGCTGTCACTATCG	TTGGGACTGGCTTGTTTA
Esr1	AGCATTCAAGGACACAA	CTTCCAAGTCATCTCTCTG
Fabp4	GTGGGAGTGGGCTTTGC	GCTCTTCACCTTCCTGTCGT
Fbxo32	GGCTACTGTGGAAGAGACT	CAGGAGAGAATGTGGCA
Hadha	TGAAGTGTTGCTGGGGAT	CACGAATGTTCCTGCCA
Igf1	TCACACCTCTTCTACCTGGC	GTGCCCTCCGAATGCTG
Irs2	ATCAGGTATCTGGGGTGGAG	GACGGTGGTGGTAGAGGAAA
Itga3	TCATCTGTCTTCCACGGCTT	CTGGTTGAGGACTGGGTAGG
Itga5	AAGGGAGAGGAGCCTGTGG	CGGGTGAAGTTTTCTGTGGA
Jun	GCCCCTGTCCCCTATCG	TGAGTTGGCACCCACTGTTA
Junb	TCACGACGACTCTTACGCAG	GACCCTTGAGACCCCGATAG
Lipe	TGAGATTGAGGTGCTGTCGT	GGTAACTGTGAGCCTGGGAT
Pik3c2a	AGCCCACCATTCGTTTCC	GCTTCAGCATCTGTAGTTTG
Pik3ca	CCTGGGGAAACATAAACTT	AAACTTCACCACACTGCTG
Slc27a1	GGAGTCGTGGAGGTCTGAAG	GATGATTGATGGTTGCCGC
Tnnc1	AGGTGATGAGGATGCTGG	ACTTCCCTTTGCTGTCGTC
Tnni1	TGTCTCTCAGTGCCCTTCA	ATCTCTCTGGTGTTGTGGA
Tnnt1	GCACTAAAAGACCGCATTG	AGTTTCATCTCCCGACCAG
Trim63	TAGCCTGATTCCTGATG	GGTCCAGTAGGGATTCG

Supplemental Table 3. qPCR primers used to identify GR enrichment at putative binding sites

Category	Gene of Interest	Location	Forward Primer (5' to 3')	Reverse Primer (5' to 3')
negative control	n/a	chr15:15,752,358-15,752,522	GCCGAAATGTATGAGTAGCCA	AATGAATGAGCCCTTCCCCA
positive control	*Fkbp5*	chr17:28,420,152-28,420,344	GCCACATTCAGAACAGG	TACTCCAACAAACCCCAC
calcium-handling	*Cacna1h*	chr17:25,434,130-25,434,246	CACGCCTGCTGAGCCCCG	TCCTTCCCCACCCCCACTGC
calcium-handling	*Tnnc1*	chr14:31,206,433-31,206,508	GCAGAACCTTCCACGCACT	CGACCCAGGGGCTTTGA
calcium-handling	*Tnnt1*	chr7:4,522,438-4,522,594	TAGAGTCAAAGGAGGAGGGG	GACACTGAGATAAGGGGCGA
IGF1 pathway	*Akt1*	chr12:112,666,788-112,666,871	CCTTTACCCTCTAAGCCATCT	TTACCCATCCTCCCTCTCC
IGF1 pathway	*Ar*	chrX:98,148,575-98,148,652	CAACCATACTACGCCAGCAC	TTTCCTTTTCTCCCCTCCC
IGF1 pathway	*Pik3c2a*	chr7:116,444,010-116,444,188	CCTCTCCTCCGACAGTTAC	GCCAGACATCACACCCAG
IGF1 pathway	*Pik3ca*	chr3:32,454,532-32,454,665	GCACGCTGCTGTCTTTGT	ATAATACCCCAGTTCCCCA
lipid metabolism	*Cidea*	chr18:67,511,497-67,511,622	TTACTCTTCCCCACTTATGAT	CTGTCTGTGTCTGCTGATGT
lipid metabolism	*Cidec*	chr6:113,385,708-113,385,789	TGGGTTCTGGAATGTGGT	TAGGGTGAAGTCTCTGGC
lipid metabolism	*Esr1*	chr10:5,674,439-5,674,518	GGAACACTGGTGAAGGCT	ATGCTCTCTTTTAGTATTATTTTA
lipid metabolism	*Slc27a1*	chr8:71,561,278-71,561,409	ATTACTCTTTGAGGGGACAT	AAGGGAGTAGTGGGGGAA
lipid metabolism	*Slc27a1*	chr8:71,575,870-71,575,940	CAGGAGGCAGAGACAGGC	TAGAACTTGCTACATAGACCAGG

Supplemental Table 4. Abundance of compounds of interest from untargeted lipidomics

Compound	Compound Class	Vehicle					Weekly					Fold Change	P-value
CL 78:12	cardiolipin	2.14	2.88	2.59	0.86	3.03	5.53	2.44	3.23	4.17	5.27	1.79	0.0324
DG 12:0/18:2	diglyceride	9.79	7.98	8.99	8.33	10.11	7.28	7.99	6.98	8.40	7.99	0.85	0.0267
DG 14:0/16:1	diglyceride		16.53	16.44	15.46	16.97	15.87	0.12	12.66	1.45	0.03	0.37	0.0323
DG 18:0/18:1	diglyceride	2.48	1.86	2.08	1.68	2.59	1.25	1.68	1.73		1.78	0.75	0.0520
DG 18:1/18:1	diglyceride	3.06	1.96	3.36	2.24	3.17	2.08	1.62	1.84		1.89	0.67	0.0270
DG 18:1/22:6	diglyceride	2.43	1.70	2.08	1.15	2.27	1.16	1.35	1.29		1.45	0.68	0.0540
LPC 19:1	lyso-phosphatidylcholine	3.23	0.24	-0.51	-0.24	2.37	-0.43	-0.83	-18.6	-5.44	-22.1	-9.33	0.0528
PC 13:0/20:4	phosphatidylcholine	0.72	0.06	-0.37	-2.20	0.52	-0.06	-0.11	4.81	4.95	3.72	-10.50	0.0487
PC 14:0/16:2	phosphatidylcholine	9.79	8.83	9.07	7.10	9.55	9.50	9.25	11.31	10.42	12.12	1.19	0.0507
PC 16:0/16:1 RT: 6.592	phosphatidylcholine	1.43	-0.52	-0.81	0.52	2.21	2.62	2.33	4.24	3.42	2.30	5.25	0.0076
PC 16:0/16:1 RT: 7.634	phosphatidylcholine	3.23	-13.1	-13.3	-0.10	-12.40	2.63	2.03	2.82	3.53	2.30	-0.37	0.0263
PC 16:0/18:0	phosphatidylcholine	-10.0	-10.6	-10.9		-9.95	5.93	5.01	5.29	4.96		-0.51	<0.000001
PC 17:0/22:6	phosphatidylcholine	-17.9	1.20	0.79	-20.7	-17.8		1.16	2.96	2.20	0.87	-0.16	0.0561
PC 18:0/20:4	phosphatidylcholine	6.22	13.87	6.14	4.94	-4.94	13.85		14.34	14.92	13.22	2.69	0.0360
PC 18:1/20:5	phosphatidylcholine	0.34	-0.16	-1.22	-0.32	0.16	-0.52	1.41	0.92	1.52	1.74	-4.23	0.0335
PC 19:1/18:2	phosphatidylcholine	0.09	0.29	-0.25	-0.80	-0.09	0.14	1.48	1.80	1.35	0.46	-6.84	0.0114
PE 38:7	phosphatidylethanolamine	6.55	6.04	6.14		6.54	7.77	7.36	7.26	6.08	7.45	1.14	0.0411
PE 38:8	phosphatidylethanolamine	-0.72	-0.33	-0.70		-0.92	0.77	-0.09	0.15	-0.46	-0.23	-0.04	0.0323
PEtOH 16:0/18:3	phosphatidylethanol	0.56	0.01	11.43	12.82	16.95	-0.01	-0.20	0.64	0.86	-0.33	0.02	0.0443
TG 16:0/18:2/22:6	triglyceride		0.59	0.54	0.44	0.58	0.53	0.27	0.30	0.30	0.39	0.67	0.0225
TG 18:0/18:1/18:2 RT: 5.751	triglyceride	16.14	17.66	14.68	14.81	13.88		0.42	0.42	0.36	0.40	0.03	<0.000001
TG 18:0/18:1/18:2 RT: 5.752	triglyceride	16.26	17.66	14.67	14.81	15.09	0.48	0.42	0.42	0.36		0.03	<0.000001
TG 18:1/18:1/22:4	triglyceride	6.31	2.81		4.59	5.13	1.81	2.23	1.29	4.62	2.96	0.55	0.0531
TG 18:2/22:6/22:6	triglyceride	-0.52	-1.54	-2.97		-3.78	-3.42	-14.4	-14.4	-14.5	-2.84	4.51	0.0471
TG 56:8	triglyceride		0.59	0.54	0.44	0.58	0.51	0.27	0.30	0.30	0.39	0.66	0.0156
TG 62:14	triglyceride	-0.51	-1.85	-2.98		-3.78	-3.43	-14.4	-14.4	-14.5	-2.85	4.36	0.0484

RT: retention time

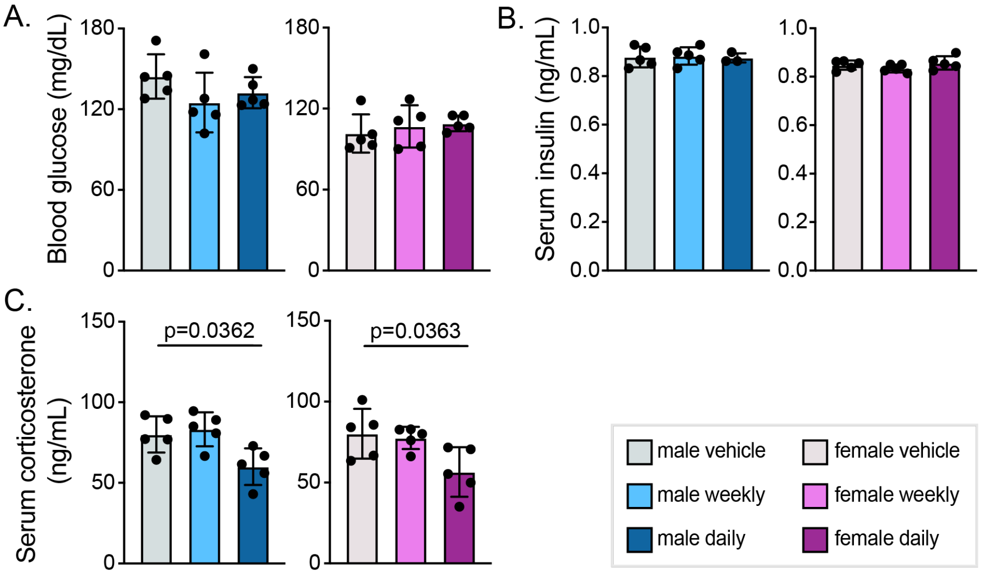

Supplemental Figure 1. Circulating glucose, insulin, and corticosterone were not changed by weekly prednisone. (A-B) Blood glucose (A) and insulin (B) did not change in response to weekly or daily treatment in male or female mice. **(C)** Daily-treated animals of both sexes have reduced circulating corticosterone, but weekly-treated animals did not in comparison to vehicle-treated. (C) One-way ANOVA.

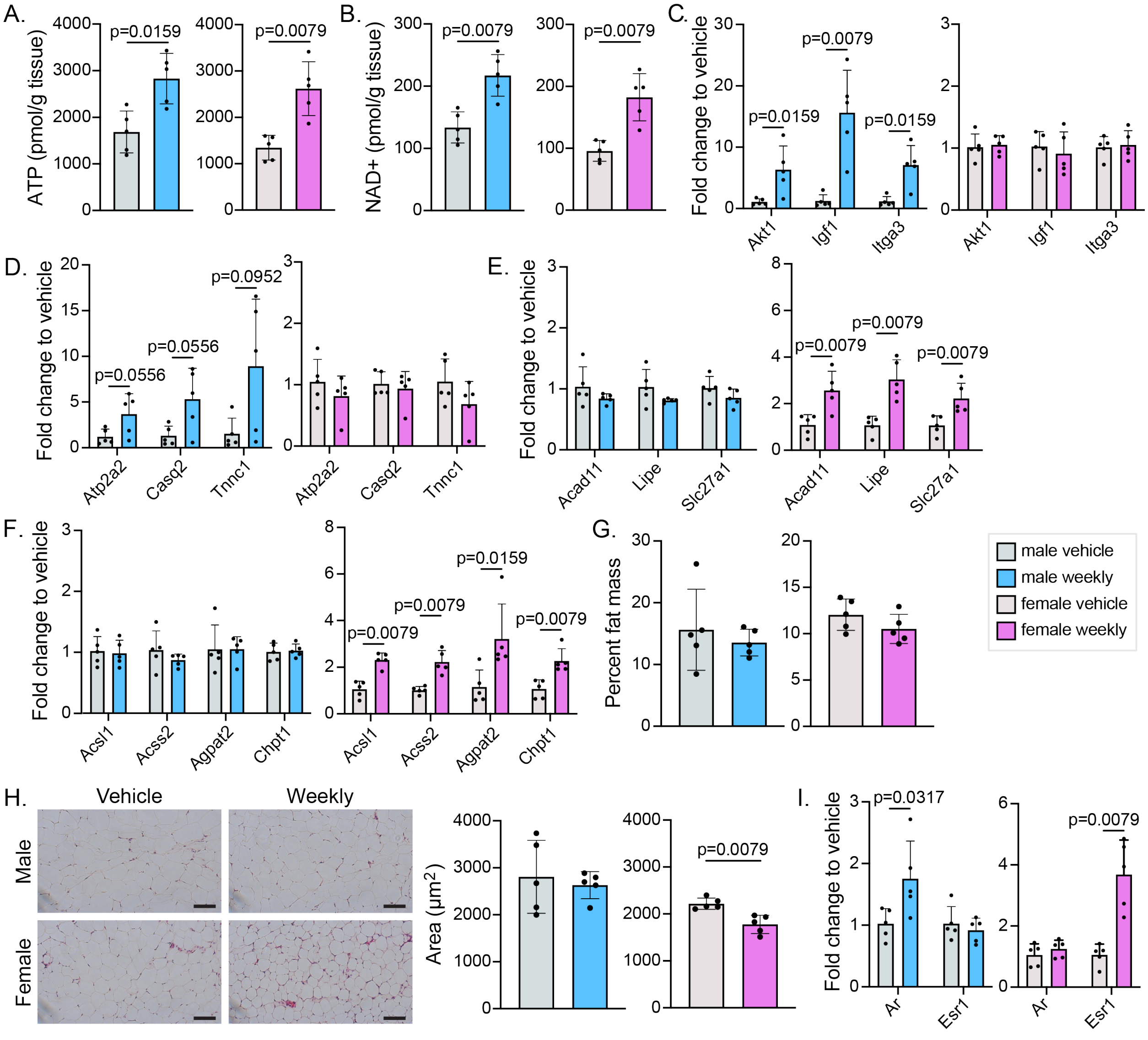

Supplemental Figure 2. Weekly prednisone exerts the same effect in 18-week-old mice as 10-week-old. (A-B) Weekly-treated mice had increased concentrations of ATP (A) and NAD+ (B) compared to vehicle-treated animals. **(C-F)** Animals administered weekly prednisone starting at 18 weeks had sex-specific upregulation of IGF1 pathway (C), calcium-handling (D), and lipid metabolism (E-F) genes. **(G-H)** Although whole body percent fat mass did not change after four weeks of treatment (G), visceral fat pad adipocytes had significantly reduced cross-sectional area in females (H). **(I)** 18-week-old mice had sex-specific upregulation of the genes encoding the sex steroid receptors. (A-I) Mann-Whitney; black bar = 100μm.

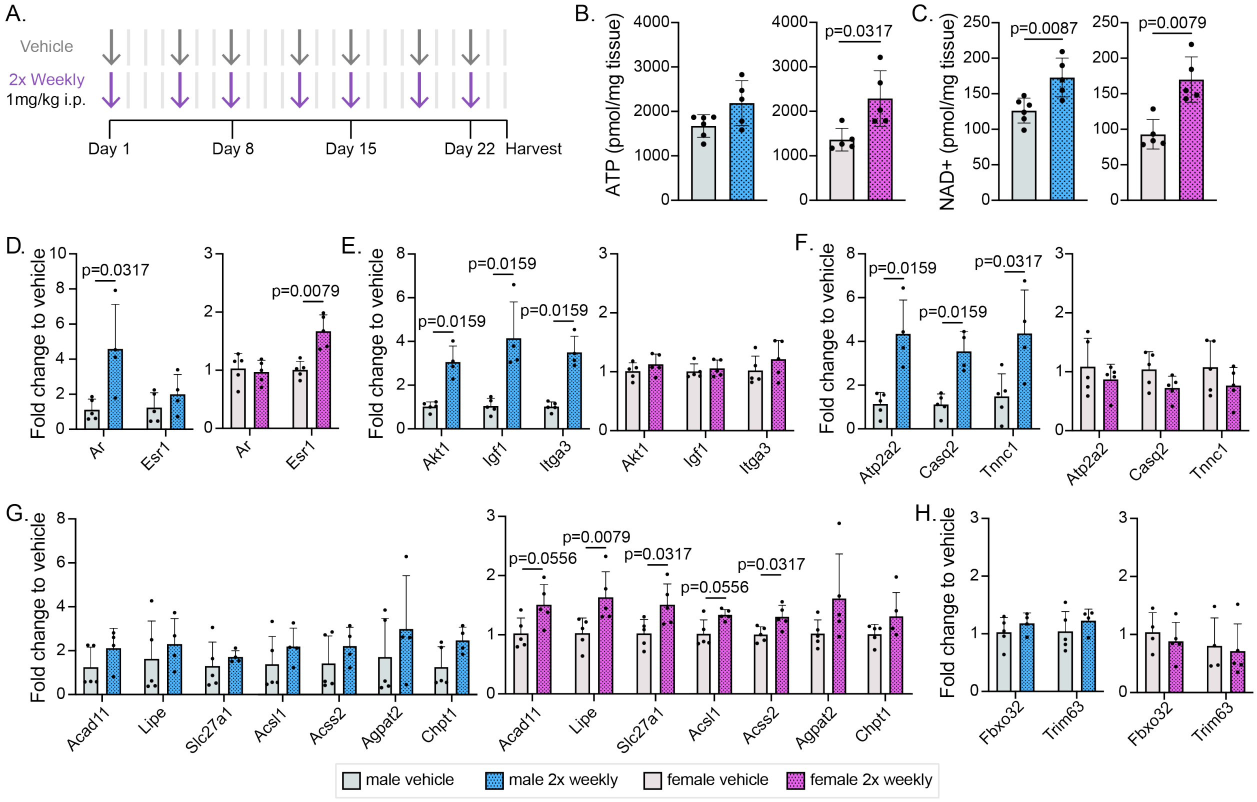

Supplemental Figure 3. Twice-weekly prednisone exerts some of the same effects as once-weekly. (A) C57BL/6 mice were treated for three weeks with vehicle (DMSO) or prednisone twice a week and then analyzed. Arrows indicate i.p. injection; bars indicate no injection. **(B-C)** Female mice treated twice-weekly had increased concentrations of ATP (B) and NAD+ (C) compared to vehicle-treated animals, while males only exhibited increased NAD+. **(D-H)** Animals administered twice-weekly prednisone had sex-specific upregulation of sex steroid receptor (D), IGF1 pathway (E), calcium-handling (F), and some lipid metabolism (G) genes. **(H)** Atrogene expression was not upregulated by twice-weekly prednisone. (A-H) Mann-Whitney

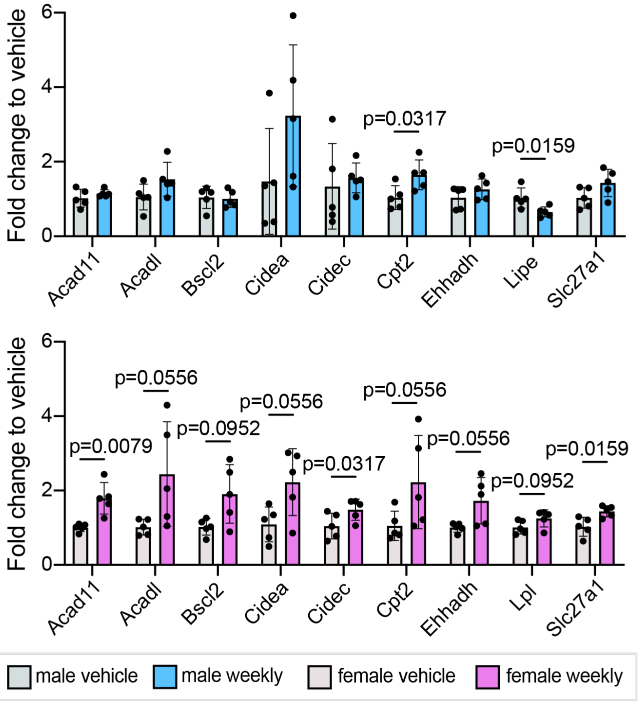

Supplemental Figure 4. qPCR validation of lipid metabolism genes. Lipid metabolism genes were significantly upregulated in weekly-treated females but were mostly unchanged in weekly-treated males. P-value determined by Mann-Whitney.

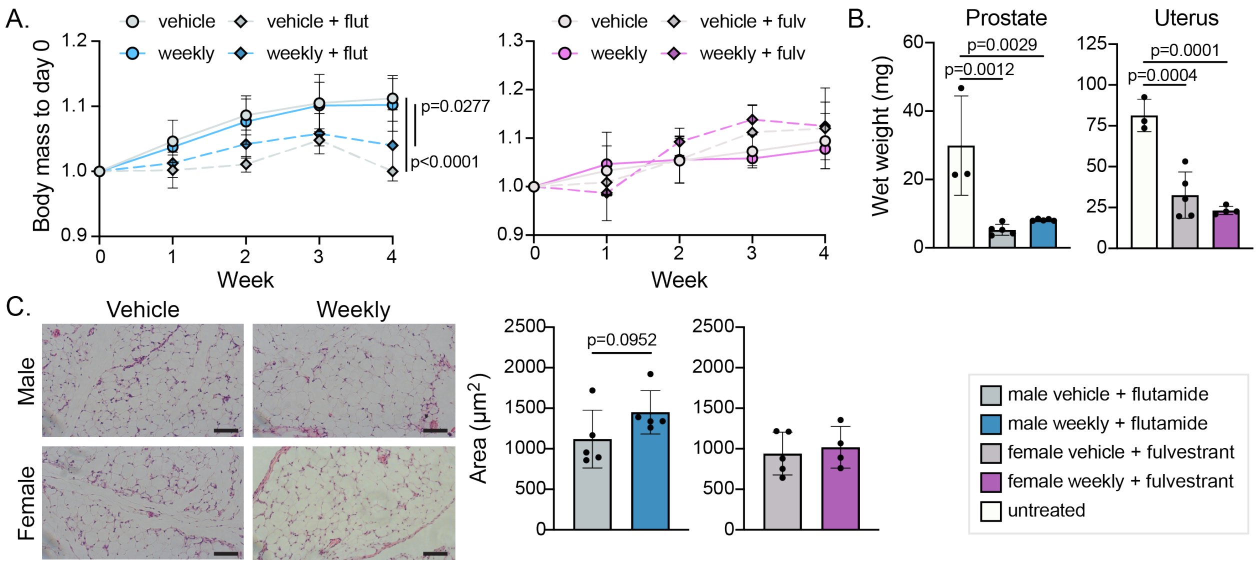

Supplemental Figure 5. Sex steroid receptor inhibition affects body mass and reproductive organ size. (A) Male mice treated with flutamide for four weeks had significantly reduced body weight, while females treated with fulvestrant had no change in body mass. **(B)** Both male and female mice had significantly reduced reproductive organ wet weight after four weeks of sex steroid inhibitor treatment. **(C)** Visceral fat pad adipocytes had significantly increased cross-sectional area in males after co-treatment with flutamide and weekly prednisone, while female adipocytes did not change in size. (A) Two-way ANOVA; (B) One-way ANOVA; (C) Mann-Whitney; black bar = 100μm

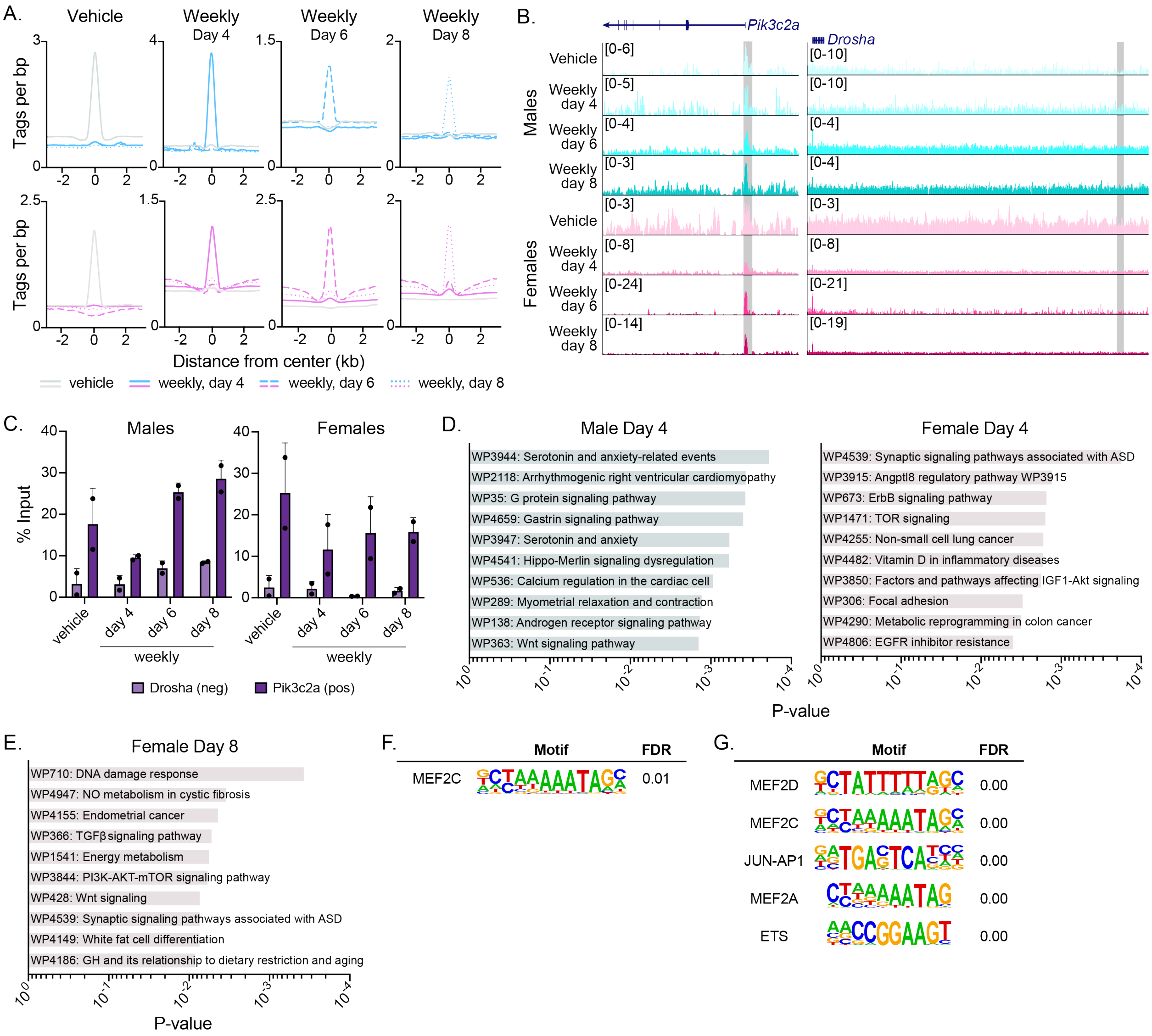

Supplemental Figure 6. H3K27ac ChIPseq in isolated myofibers. **(A)** Tag density distribution plots for peaks unique to each time point; top row = males, bottom row = females. **(B-C)** ChIPseq tracks (B) and qPCR validation (C) of loci called as either a peak at all time points (*Pik3c2a*) or not called as a peak at any time point (*Drosha*). **(D)** Gene ontology of top 500 enhancers by peak score in male and female myofibers four days after last prednisone injection. **(E)** Gene ontology of top 500 enhancers by peak score that were maintained out to day eight in females. **(F-G)** Most significantly enriched motifs in male (F) and female (G) enhancers that were maintained out to day eight after last injection.
